# Use of social health insurance for hospital care by internal migrants in China—Evidence from the 2018 China migrants dynamic survey

**DOI:** 10.3389/fpubh.2022.1008720

**Published:** 2022-11-24

**Authors:** Qiang Yao, Hanxuan Li, Chaojie Liu

**Affiliations:** ^1^School of Political Science and Public Administration, Wuhan University, Wuhan, China; ^2^Centre for Social Security Studies, Wuhan University, Wuhan, China; ^3^School of Psychology and Public Health, La Trobe University, Melbourne, VIC, Australia

**Keywords:** migrant, social health insurance, health service, China, Heckman model

## Abstract

**Background:**

China's welfare system including social health insurance has been closely linked to its unique household registration system, despite high population mobility over the past few decades. This study aimed to determine the pattern of health insurance usage from internal migrants in mainland China for hospital care.

**Methods:**

Data were extracted from the 2018 China Migrants Dynamic Survey. The respondents who enrolled in a social health insurance program and reported illness or injury over the past year were eligible for this study (*n* = 15,302). Two groups of outcome indicators were calculated assessing the use (incidence and settlement location) of insurance funds for hospital care and the burden of hospital expenditure (total hospital expenditure, out-of-pocket payments, and share of insurance reimbursement), respectively. Logit regression and Heckman's sample selection models were established to determine the predictors of insurance fund usage and the burden of hospital expenditure, respectively.

**Results:**

Most respondents enrolled in a social health insurance program outside of their residential location (70.72%). About 28.90% were admitted to a hospital over the past year. Of those hospitalized, 72.98% were admitted to a hospital at their migration destination, and 69.96% obtained reimbursement from health insurance, covering on average 47% of total hospital expenditure. Those who had a local insurance fund aligned with residency (AOR = 2.642, 95% CI = 2.108–3.310, *p* < 0.001) and enrolled in employment-based insurance (AOR = 1.761, 95% CI = 1.348–2.301, *p* < 0.001) were more likely to use insurance funds for hospital care, and paid less out-of-pocket (β = −0.183 for local funds, *p* = 0.017; β = −0.171 for employment-based insurance, *p* = 0.005) than others. A higher share of insurance reimbursement as a proportion of hospital expenditure was found in the employment-based insurance enrollees (β = 0.147, *p* < 0.001). Insurance claim settlement at the residential location was associated with lower total hospital expenditure (β = −0.126, *p* = 0.012) and out-of-pocket payments (β = −0.262, *p* < 0.001), and higher share of insurance reimbursement (β = 0.066, *p* < 0.001) for hospital expenditure.

**Conclusion:**

Low levels of health insurance benefits for hospital care are evident for internal migrants in mainland China, which are associated with the funding arrangements linked to household registration and inequality across different funds.

## Introduction

There has been a consensus that high population mobility has contributed significantly to the unprecedented economic growth in China (1, 2). However, China has still maintained its unique household registration system, also known as “Hukou” originally designed for limiting population mobility. Those who work or live outside of their household registration location were referred to as internal migrants (3). In 2021, the internal migrant population reached 384.67 million, accounting for 27.3% of the entire population in China (4). Compared to the long-term permanent local residents, the internal migrants face great challenges in accessing health care services due to barriers resulting from low socioeconomic status and the Hukou-linked social welfare arrangements (5, 6). A previous study found that more than one-third of internal migrants in China do not follow advice from doctors for hospital admissions (7). On the one hand, internal migrants are often exposed to higher health risks compared to their local counterparts by taking labor-intensive low-paid jobs such as construction, transportation, manufacturing, and catering (8). On the other hand, they experience a loss of social capital from their Hukou location (9) and face discrimination in job opportunities and social welfare entitlements in their migration destination (10).

Social health insurance is an important tool for ensuring accessibility of health care (11) and preventing financial risks associated with health care services (12). Empirical evidence shows that health insurance can not only increase the use of medical care services (13, 14), such as medical consultations (15–19) and hospital admissions (14, 20), but also increase the use of preventive care such as physical examinations (15, 20, 21), personal health records (22), and health education (23) in the internal migrants. Health insurance can also reduce the financial burden of medical care of the internal migrants (13, 17, 19), despite increased use of healthcare services and rise in medical expenses (24–26).

China has achieved almost universal coverage of social health insurance through three major schemes: Basic Medical Insurance for Urban Employees (BMIUE), Rural New Cooperative Medical Scheme (RNCMS), and Basic Medical Insurance for Urban Residents (BMIUR). The latter two were merged in 2016 and renamed to Basic Medical Insurance for Urban and Rural Residents (BMIURR). However, social health insurance coverage of internal migrants is significantly lower than that of the rest of the population in China (27). About 10% of internal migrants do not have any health insurance (8, 28). For those covered by health insurance, the vast majority (>76%) enrolled in a program at their Hukou location (28). There have been concerns that the misalignment between health insurance fund location and the residency of enrollees may have hindered the use of health insurance (29). Previous studies showed that 80% of internal migrants paid entirely out-of-pocket for their recent medical consultations (30), and 37.1% did not have access to the on-the-spot settlement of hospital bills from their insurance funds (31). These have led to significant inequalities between the migrant and non-migrant populations (32). In addition, most internal migrants (77%) are not covered by the more generous employment-based insurance program BMIUE (8), and their insurance entitlements are seriously restricted by the financial capacity of their insurance funds (13, 33, 34). Several studies showed that unlike the BMIUE, the RNCMS failed to effectively ease the financial burden of migrant patients (19, 35, 36), including the rural-to-urban older migrants (37).

China has made great efforts in reforming its social health insurance programs. However, there is a paucity in the literature documenting evidence of the benefits of various health insurance arrangements for internal migrants at the national level. This study aimed to address the gap in the literature by analyzing the pattern of health insurance usage in internal migrants for hospital care using the 2018 China Migrants Dynamic Survey (CMDS).

## Materials and methods

### Data sources

Data were extracted from the 2018 CMDS dataset. The CMDS is an annual nationwide survey of internal migrants conducted by the National Health Commission since 2009. The survey drew samples from community residents aged 15 years and above who did not have a Hukou in their residential location (city or county), but had lived there for at least 1 month. A stratified multistage probability proportional to size (PPS) sampling strategy was adopted to select participants from all of the 31 regions/provinces in mainland China. In the first stage, 66 municipalities were identified to cover both capital and non-capital municipalities. This was followed by a selection of urban districts and rural counties in each municipality. Residential communities in each urban district and rural county (township) were subsequently identified. Finally, housing estates that accommodated migrants explicitly (e.g., factory dormitory) and inexplicitly (e.g., residential committee) were selected. All of the migrants residing in the selected housing estates were invited to participate in the study. This resulted in a total of 152,000 study participants in the 2018 CMDS. Further details of the sampling process have been published elsewhere (38).

Data were collected through household visits, tapping into the household structure, sociodemographic characteristics (age, gender, education, employment, income), health status (self-rating), health insurance enrolments, and use of healthcare services of the respondents (one member only from each household).

**Figure 1:**
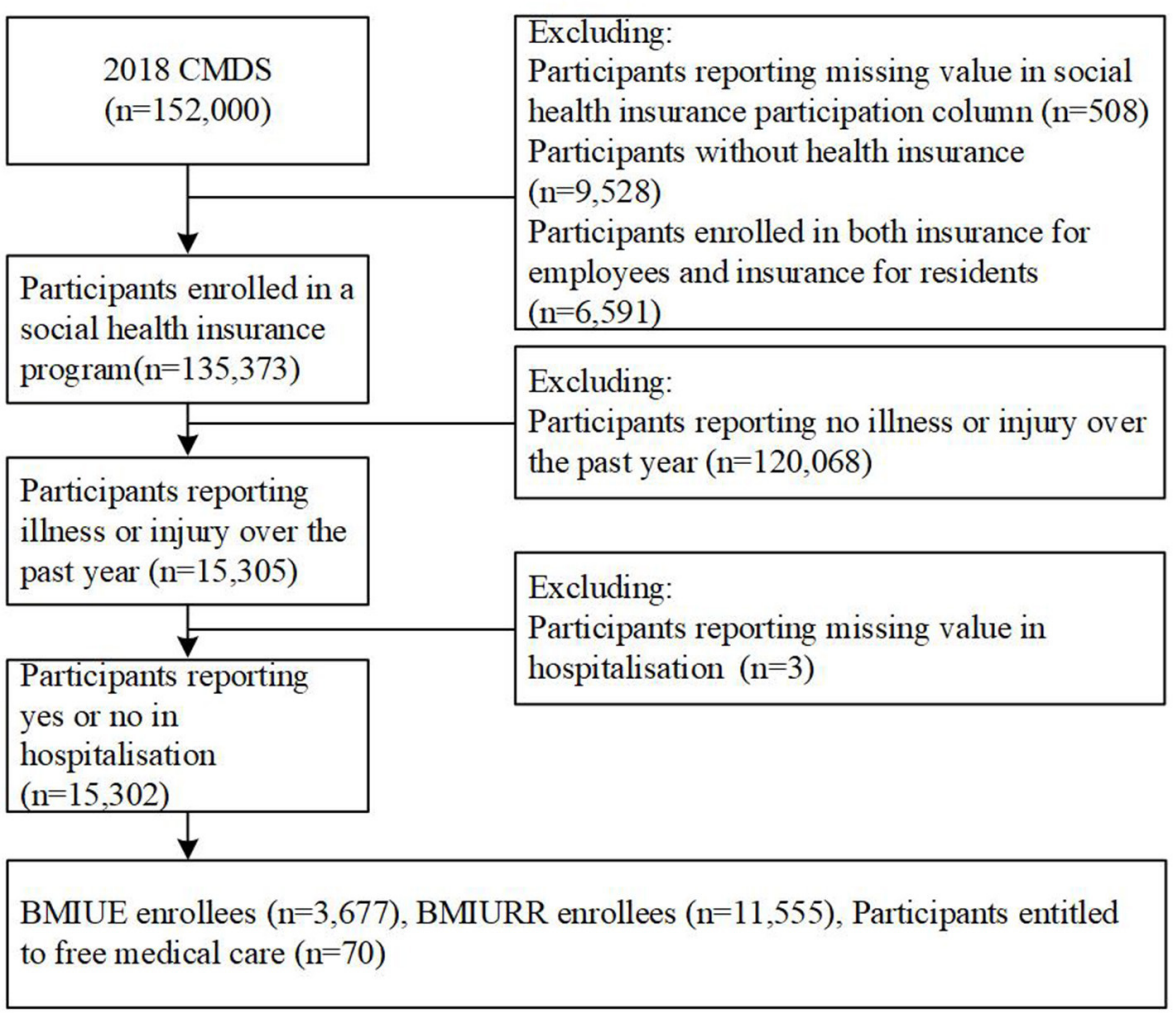
Screening flowchart of study participants.

Eligible participants for the current study were restricted to those who enrolled in a social health insurance program and reported illness or injury over the past year. Those who did not enroll in any insurance, had missing values in insurance enrolments, and enrolled in both BMIURR and BMIUE were excluded (Figure 1). Overall, 135,280 (89%) of the 2018 CMDS respondents were eligible for this study. Of the eligible respondents, 15,302 (11.3%) reported hospital admissions over the past year and were included in the final data analyses.

### Theoretical framework

**Figure 2:**
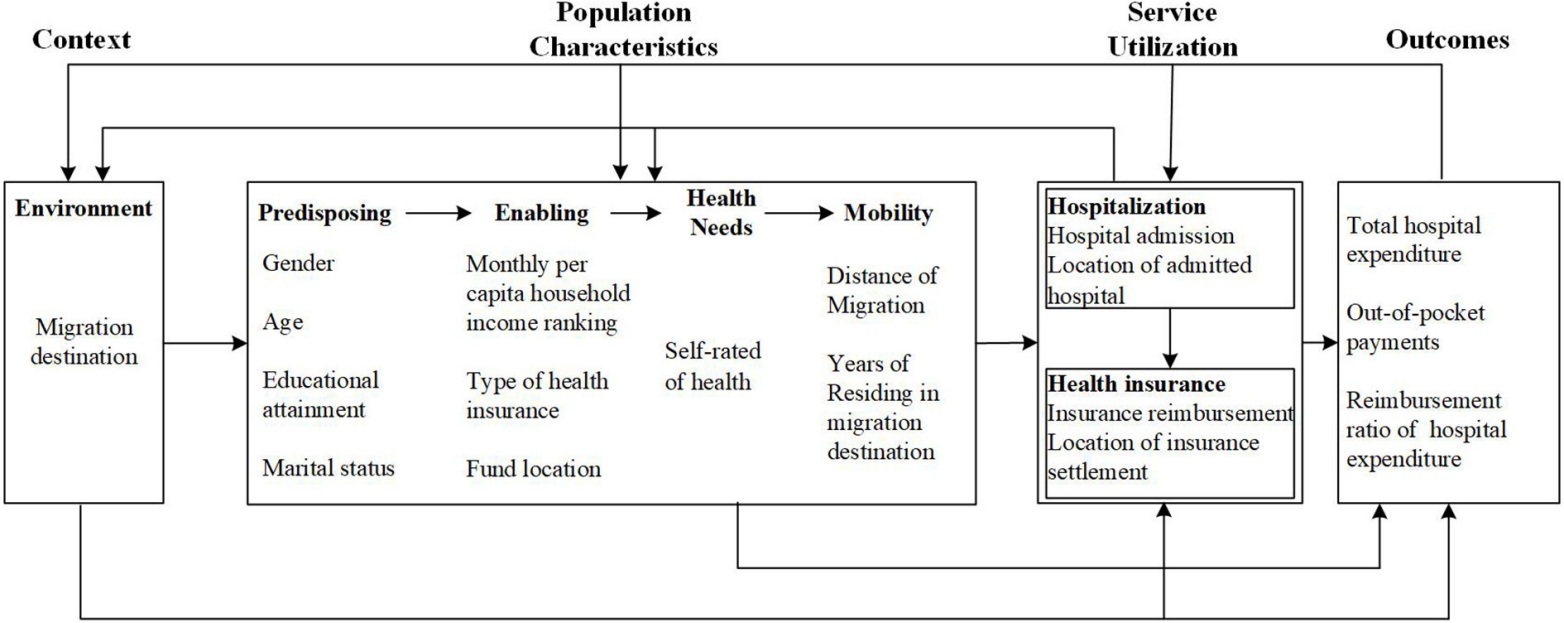
Theoretical framework of the study.

The Andersen's health service utilization model guided the analytical framework in this study. The Andersen's model has been widely recognized as one of the best for explaining and predicting healthcare service behaviors (39, 40). Five broad factors (environment, predisposing, enabling, health needs, and mobility) were measured as predictors of hospital admissions in this study in line with the Andersen's model (Figure 2).

### Data analysis

#### Dependent variables

Three groups of outcome indicators (dependent variables) in relation to hospital care were calculated. In China, social health insurance prioritizes coverage of hospital services (41). In Anhui, for example, the BMIURR reimburses 60–85% of hospital expenses, compared with 55% for outpatient care (42). On-the-spot settlement of medical expenditure across provinces is also focused on hospital expenses, except in a few regions (Yangtze River Delta, Beijing-Tianjin-Hebei, Yunnan-Guizhou-Sichuan-Chongqing-Tibet) that have recently (since 2019) expanded it to cover outpatient care (43).

(1) Hospital care usage: the 2018 CMDS asked respondents who reported illness or injury over the past year: “Were you ever admitted to a hospital over the past year?” (yes or no) and “Where were you hospitalized in the most recent hospital admission?” (residential location or non-residential location). We calculated the hospitalization rate of the study participants over the past year and the percentage share of local (residential location) hospitalization in the most recent admission of those hospitalized.

(2) Use of health insurance in hospital care: the 2018 CMDS asked the respondents who were admitted to hospitals: “Were you ever reimbursed from health insurance for your hospital expenditure over the past year” (yes or no) and “Where was your most recent insurance claim settled?” (residential location or non-residential location). We calculated the percentage of hospital admissions that were subsidized (partially or totally) by insurance funds and the percentage share of local (residential location) insurance settlements in those who enjoyed insurance reimbursement.

(3) Share of health insurance reimbursement in hospital expenditure: the 2018 CMDS asked the respondents who were admitted to hospitals: “How much was the total expenditure of your most recent hospitalization?” and “How much did you pay out-of-pocket for the most recent hospital care on top of those reimbursed and deducted from your individual medical saving account?”. We calculated the share of health insurance reimbursement as a proportion of total hospital expenditure.

#### Independent variables

The use of health insurance is a major interest of this study. We assessed the pattern of health insurance usage through two indicators: type of insurance (BMIUE, BMIURR, free medical care) and insurance fund location (residential location vs. non-residential location).

#### Control variables

The Andersen's healthcare utilization model guided the selection of control variables in reference to previous studies (31, 33, 37, 44). The environmental factor was measured by the economic zone of migration destination classified by the Chinese government: Pearl river delta; Yangtze river delta, Circum-Bohai sea; and others. The predisposing factor was measured by gender (male vs. female), age (years), educational attainment (primary school or below, junior high school, and senior high school and above), and marital status (single, married, or cohabiting, and divorced or widowed). The enabling factor was measured by monthly per capita household income (ranked in quintile). The health needs factor was measured by self-rated health (poor, general, and good). The mobility factor was measured by the distance of migration (inter-county, inter-city, and inter-province), and years of residing in the migration destination.

#### Statistical analysis

The characteristics of study participants were described using frequency distributions and were compared between the BMIURR and BMIUE enrollees. The percentages of study participants hospitalized and their health insurance usage for hospital care were calculated and compared between those with (residential) local and non-local insurance funds through Chi-square tests. Median values and interquartile ranges (IQR) of the hospital expenditure and means and standard deviations (SD) of the percentage share of insurance reimbursement in hospital expenditure were calculated and compared between those with (residential) local and non-local insurance funds by the type of insurance programs through two-sample Wilcoxon rank-sum (Mann-Whitney) tests and student *t*-tests, respectively.

Logit regression models were established: (1) for hospitalization rate using the entire sample with different types of health insurance programs; (2) for the percentage share of (residential) local hospitalization and the percentage share of insurance-subsidized hospital admissions using the sub-sample of hospitalized participants with different types of health insurance programs; (3) for the (residential) local settlement of insurance claims and the reimbursement ratio of hospital expenditure using the sub-sample of participants who were subsidized by different insurance funds for hospital care. We also performed subgroup analyses by household income (in quintiles: lowest, lower, middle, high, highest) to test the robustness of the modeling results.

The sample selection model proposed by Heckman (45) was adopted to establish the models predicting total hospital expenditure (natural logarithm transformed) and the share of insurance reimbursement in hospital expenditure. The Heckman model allowed us to use the entire sample, rather than the sub-sample of hospitalized patients, to address the endogeneity problem arising from sample selection: some unobservable confounders related to hospital admissions may be linked to the error terms of health insurance usage for hospital care in the modeling. The Heckman's approach involved two steps. A selection model was developed first.


$$p_{i}^{*}\;=\;\alpha +\beta x_{i}+\mu_{i},p_{i}\;=\;\left\{\begin{array}{c} 1\;\begin{array}{c} if\;p_{i}^{*}>0 \end{array}\\ 0\;\begin{array}{c} if\;p_{i}^{*}\leq 0 \end{array} \end{array}\right\}$$

In the formula, $p_{i}^{*}$ indicates the probability of the event occurrence (hospital admission or insurance reimbursement). **p**_**i**_ represents the observed individual behavior (hospital admission or insurance reimbursement). **x**_**i**_ refers to the independent and control variables. **α** is the intercept term. **β** is the parameters to be estimated. **μ**_**i**_ is the random disturbance term.

The selection model enabled the estimation of **λ**_**i**_, the Mills rate, which denotes the ratio of the cumulative distribution function to the density function.

The second step estimated the outcome indicators (**y**_**i**_**)** relating to hospital expenditure.


$$y_{i}\;=\;\omega +\theta z_{i}+\gamma {\text{λ}}_{i}+\varepsilon_{i}$$

In the formula, **y**_**i**_ represents total hospital expenditure (natural logarithm transformed), out-of-pocket payments for hospital care (natural logarithm transformed), or percentage share of insurance reimbursement in hospital expenditure. **λ**_**i**_ controls for the heterogeneity that leads to the sample selection bias. **z**_**i**_ refers to the independent variables and control variables, which should exclude at least one variable in **x**_**i**_ (45, 46). **θ** is the parameters to be estimated. **ω** is the intercept term.

We tested collinearity of the independent variables using variance inflation factor (VIF). A VIF value of higher than 5 or a tolerance below 0.2 is generally accepted as an indication of high multicollinearity that can negatively impact the regression model. Although none of the independent variables had a VIF exceeding 5 (Supplementary Table S1), we still established two types of modeling in line with the recommendations from Vatcheva et al. (47): one including “local fund” and “location of insurance settlement”, respectively; another including both.

All analyses were performed using STATA version 16.0 (SE) for Windows (Stata Corp LLC, College Station, TX, USA). Missing values were treated through pairwise deletion. A two-sided *p*-value of < 0.05 was considered statistical significance.

## Results

### Characteristics of study participants

More than half (57.08%) of the study participants who reported illness or injury were female. Most were younger than 60 years (89.73%). The vast majority received junior high school or above education (74.95%), were married or cohabiting (85.52%) at the time, self-rated general or good health (86.67%), migrated across cities or provinces (81.34%), and resided in the migration destination for more than 1 year (86.80%). Nearly four in ten migrated to the three major economic development zones in mainland China: Pearl River Delta, Yangtze River Delta, and Bohai Rim. Over 75% of study participants enrolled in BMIURR, and 70.72% enrolled in a health insurance program outside of their residential location.

Compared with the BMIUE enrollees, the BMIURR enrollees were older (*p* < 0.001), had lower education (*p* < 0.001), earned lower income (*p* < 0.001), and were more likely to migrate across counties (*p* < 0.001) but less likely to have a local insurance fund (*p* < 0.001) (Table 1).

**Table 1:** Characteristics of study participants (*n* = 15,302).

**Variable**	**Coding of value**	**Sample size**		**BMIURR**		**BMIUE**		***P***
		***n***	**%**	***n***	**%**	***n***	**%**	
Gender	0 = female	8,735	57.08	6,759	58.59	1,951	57.18	< 0.001
	1 = male	6,567	42.92	4,796	41.51	41.51	46.94	
Age (years)	1 = 15–29	4,060	26.53	2,913	25.21	1,140	31.00	< 0.001
	2 = 30–44	5,750	37.58	4,142	35.85	1,598	43.46	
	3 = 45–59	3,921	25.62	3,375	29.21	535	14.55	
	4 = 60 and above	1,571	10.27	1,125	9.74	404	10.99	
Educational attainment	1 = Primary school or below	3,833	25.05	3,567	30.87	261	7.10	< 0.001
	2 = Junior high school	5,780	37.77	4,922	42.60	845	22.98	
	3 = Senior high school and above	5,689	37.18	3,066	26.53	2,571	69.92	
Marital status	1 = Single	1,589	10.38	1,020	8.83	565	15.37	< 0.001
	2 = Married/cohabiting	13,087	85.52	10,016	86.68	3,009	81.83	
	3 = Divorced/widowed	626	4.09	519	4.49	103	2.80	
Monthly per capita household income ranking	1 = Lowest (< percentile 20)	4,300	28.10	3,873	33.52	420	11.42	< 0.001
among the survey participants	2 = Lower (percentile 20–39)	3,214	21.00	2,611	22.60	591	16.07	
	3 = Middle (percentile 40–59)	2,855	18.66	2,113	18.29	734	19.96	
	4 = Higher (percentile 60–79)	2,548	16.65	1,667	14.43	861	23.42	
	5 = Highest (≥percentile 80)	2,385	15.59	1,291	11.17	1,071	29.13	
Type of health insurance	1 = BMIURR	11,555	75.51	–	–	–	–	
	2 = BMIUE	3,677	24.03	–	–	–	–	
	3 = Free medical care	70	0.46	–	–	–	–	
Local insurance	0 = No	10,821	70.72	10,021	86.72	748	20.34	< 0.001
	1 = Yes	4,481	29.28	1,534	13.28	2,929	79.66	
Self-rated health	1 = Poor	2,137	13.97	1,873	16.21	253	6.88	< 0.001
	2 = General	4,524	29.56	3,507	30.35	983	26.73	
	3 = Good	8,641	56.47	6,175	53.44	2,441	66.39	
Distance of migration	1 = Inter-county	2,855	18.66	2,322	20.10	521	14.17	< 0.001
	2 = Inter-city	5,343	34.92	3,892	33.68	1,423	38.70	
	3 = Inter-province	7,104	46.43	5,341	46.22	1,733	47.13	
Years of residing in migration destination	1 = Below 1	2,020	13.20	1,574	13.62	443	12.05	< 0.001
	2 = 1–4	5,577	36.45	4,140	35.83	1,407	38.26	
	3 = 5–9	3,843	25.11	2,842	24.60	984	26.76	
	4 = 10 and above	3,862	25.24	2,999	25.95	843	22.93	
Migration destination	1 = Pearl River Delta	1,164	7.61	780	6.75	384	10.44	< 0.001
	2 = Yangtze River Delta	2,645	17.29	1,831	15.85	803	21.84	
	3 = Circum-Bohai Sea	2,021	13.21	1,258	10.89	745	20.26	
	4 = others	9,472	61.90	7,686	66.52	1,745	47.46	
**Total**	15,302	100.00	11,555	100.00	3,677	100.00	

BMIURR, Basic Medical Insurance for Urban and Rural Residents; BMIUE, Basic Medical Insurance for Urban Employees.

### Use of health insurance for hospital care

Almost 29% of the study participants who reported illness or injury were admitted to a hospital over the past year: 31.11% in those with a (residential) local fund compared with 27.99% in those without a local fund (*p* < 0.001). Of the hospitalized patients, 72.98% were admitted to a hospital at their migration destination; and 69.96% were subsidized by insurance funds, covering on average 47% of hospital expenditure. Again, those with a (residential) local insurance fund were more likely to be admitted locally (a gap of 19.23 percentage points, *p* < 0.001) and be subsidized by insurance funds (a gap of 20.96 percentage points, *p* < 0.001) (Table 2). Similar results were found in the subgroup analyses of participants divided by household income (in quintiles) (Supplementary Table S2).

**Table 2:** Use of health insurance for hospital care in study participants.

**Variable**	**Coding of value**	**With local insurance**		**With non-local insurance**		**Total**		***P***
		***n***	**%**	***n***	**%**	***n***	**%**	
**The entire sample (n** **=** **15,302)**								
Hospitalization	0 = No	3,087	68.89	7,792	72.01	10,879	71.10	< 0.001
	1 = Yes	1,394	31.11	3,029	27.99	4,423	28.90	
**Among those hospitalized (n** **=** **4,423)**								
Location of admitted hospital	0 = Non-residential location	193	13.85	1,002	33.08	1,195	27.02	< 0.001
	1 = Residential location	1,201	86.15	2,027	66.92	3,228	72.98	
Insurance reimbursement	0 = No	201	16.90	756	37.86	957	30.04	< 0.001
	1 = Yes	988	83.10	1,241	62.14	2,229	69.96	
**Among those with insurance reimbursement (*****n*** **=** **2,229)**								
Location of insurance settlement	0 = Non-residential location	15	1.52	743	59.87	758	34.01	< 0.001
	1 = Residential location	973	98.48	498	40.13	1,471	65.99	
**The BMIURR enrollees (*****n*** **=** **11,555)**								
Hospitalization	0 = No	968	63.10	7,273	72.58	8,241	71.32	< 0.001
	1 = Yes	566	36.90	2,748	27.42	3,314	28.68	
**Among those hospitalized (*****n*** **=** **3,314)**								
Location of admitted hospital	0 = Non-residential location	80	14.13	908	33.04	988	29.81	< 0.001
	1 = Residential location	486	85.87	1,840	66.96	2,326	70.19	
Insurance reimbursement	0 = No	84	17.54	732	40.42	816	35.63	< 0.001
	1 = Yes	395	82.46	1,079	59.58	1,474	64.37	
**Among those with insurance reimbursement (*****n*** **=** **1,474)**								
Location of insurance settlement	0 = Non-residential location	13	3.29	659	61.08	672	45.59	< 0.001
	1 = Residential location	382	96.71	420	38.92	802	54.41	
**The BMIUE enrollees (*****n*** **=** **3,637)**								
Hospitalization	0 = No	2,107	71.94	479	64.04	2,586	70.33	< 0.001
	1 = Yes	822	28.06	269	35.96	1,091	29.67	
**Among those hospitalized (*****n*** **=** **1,091)**								
Location of admitted hospital	0 = Non-residential location	111	13.50	89	33.09	200	18.33	< 0.001
	1 = Residential location	711	86.50	180	66.91	891	81.67	
Insurance reimbursement	0 = No	117	16.57	24	13.41	141	15.93	0.301
	1 = Yes	589	83.43	155	86.59	744	84.07	
**Among those with insurance reimbursement (*****n*** **=** **744)**								
Location of insurance settlement	0 = Non-residential location	2	0.34	78	50.32	80	10.75	< 0.001
	1 = Residential location	587	99.66	77	49.68	664	89.25	

BMIURR, Basic Medical Insurance for Urban and Rural Residents; BMIUE, Basic Medical Insurance for Urban Employees.

Overall, the BMIURR enrollees were less likely to be admitted to a hospital at their migration destination (70.19 vs. 81.67%), were less likely to be subsidized by insurance (64.37 vs. 84.07%), and enjoyed lower levels of insurance reimbursement (42 vs. 59%) than the BMIUE enrollees. Despite a similar level of hospitalization rate between the BMIURR and BMIUE enrollees, fund location played a different role. The BMIURR enrollees with a local fund were more likely to be hospitalized (36.90 vs. 27.42%, *p* < 0.001) and subsidized by insurance (82.46 vs. 59.58%, *p* < 0.001) than those without a local fund. Whereas, the opposite held true for BMIUE enrollees: a lower hospitalization rate was found in those with a local fund (28.06 vs. 35.96%, *p* < 0.001), and no significant differences in insurance subsidies were found (*p* = 0.301) between local and non-local funds (Table 2).

Almost all (98.48%) of the insurance-subsidized patients with a (residential) local fund settled their insurance claims locally, compared with less than half (40.13%) in those without a local fund. The median total hospital expenditure reached 8,000 Yuan, with 4,000 Yuan paid out-of-pocket. Compared with the hospitalized patients without a local insurance fund, those with a local fund had lower total hospital expenditure (0.95 times, *p* < 0.001) and out-of-pocket payments (0.65 times, *p* < 0.001), and enjoyed a higher level of reimbursement ratio (a gap of 14% points, *p* < 0.001) (Tables 2, 3). Similar results were found in the subgroup analyses of participants divided by household income (in quintiles) (Supplementary Tables S2, S3).

**Table 3:** Hospital expenses shared between insurance and out-of-pocket payments.

**Variable**	**Measurement unit**	**With local insurance**		**With non-local insurance**		**Total**		***P***
		**Median**	**Mean**	**Median**	**Mean**	**Median**	**Mean**	
		**(IQR)**	**(SD)**	**(IQR)**	**(SD)**	**(IQR)**	**(SD)**	
**The sub-sample of participants who were subsidized (*****n*** **=** **2,186)**								
Total hospital expenditure	Yuan	7,600		8,000		8,000		< 0.001
		(6,400)		(8,000)		(7,000)		
Out-of-pocket payments	Yuan	3,000		4,600		4,000		< 0.001
		(3,500)		(5,500)		(5,000)		
Reimbursement ratio of hospital	%		0.55		0.41		0.47	< 0.001
expenditure			(0.21)		(0.23)		(0.23)	
**The sub-sample of participants who were subsidized by BMIURR (*****n*** **=** **1,466)**								
Total hospital expenditure	Yuan	6,800		8,000		8,000		< 0.001
		(7,900)		(7,000)		(7,100)		
Out-of-pocket payments	Yuan	3,000		4,800		4,200		< 0.001
		(4,500)		(5,200)		(5,600)		
Reimbursement ratio of hospital	%		0.50		0.39		0.42	< 0.001
expenditure			(0.22)		(0.22)		(0.22)	
**The sub-sample of participants who were subsidized by BMIUE (*****n*** **=** **711)**								
Total hospital expenditure	Yuan	8,000		10,000		8,000		< 0.001
		(6,000)		(13,000)		(6,858)		
Out-of-pocket payments	Yuan	3,000		4,000		3,000		0.001
		(3,400)		(6,000)		(3,400)		
Reimbursement ratio of hospital	%		0.59		0.59		0.59	0.980
expenditure			(0.19)		(0.21)		(0.20)	

BMIURR, Basic Medical Insurance for Urban and Rural Residents; BMIUE, Basic Medical Insurance for Urban Employees.

Of those without a local fund, the BMIURR enrollees were less likely to settle insurance claims locally (38.92 vs. 49.68%), less likely to be subsidized by insurance (59.58 vs. 86.59%), and paid more out-of-pocket (4,200 vs. 3,000 Yuan) than the BMIUE enrollees, despite similar total hospital expenditure. Local funds were associated with lower total hospital expenditure (0.85 times, *p* < 0.001), lower out-of-pocket payments (0.625 times, *p* < 0.001), and higher reimbursement ratio (a gap of 11 percentage points, *p* < 0.001) in the hospitalized BMIURR enrollees. Similarly, local funds were associated with lower total hospital expenditure (0.80 times, *p* < 0.001) and lower out-of-pocket payments (0.75 times, *p* = 0.001) in the hospitalized BMUE enrollees (Tables 2, 3).

### Predictors of hospitalization and use of health insurance for hospital care

Higher odds of hospitalization were found in those who were married (AOR = 4.375–6.062, 95% CI = 3.367–7.253, *p* < 0.001) and had higher educational attainment (AOR = 1.190–1.376, 95% CI = 1.073–1.549, *p* < 0.01). Whereas, lower odds of hospitalization were associated with male gender (AOR = 0.633, 95% CI = 0.585–0.684, *p* < 0.001), older age (AOR = 0.439–0.641, 95% CI = 0.387–0.754, *p* < 0.001), better self-rated health (AOR = 0.500–0.593, 95% CI = 0.444-0.670, *p* < 0.001), and longer distance of migration (AOR = 0.701–0.896, 95% CI = 0.630–0.992, *p* < 0.05). Having a local fund was a significant predictor of higher odds (AOR = 1.239, 95% CI = 1.118–1.372, *p* < 0.001) of hospitalization, although the type of insurance was not a significant predictor after adjustment for variations in other variables (Table 4). Similar results were found in the subgroup analyses of study participants divided by household income (in quintiles), although local funds were not a significant predictor of hospitalization rate in those with higher income (Supplementary Table S4a).

**Table 4:** Health insurance use for hospital care: results of logit regression models.

**Variable**	**Hospitalization** **(*****n*** = **15,302)**				**Local (residential) admission (*****n*** = **4,423)**				**Insurance reimbursement (*****n*** = **3,186)**				**Local (residential) settlement of insurance claims (*****n*** = **2,229)**			
	**OR**	***P***	**95% CI**		**OR**	***P***	**95% CI**		**OR**	***P***	**95% CI**		**OR**	***P***	**95% CI**	
**Type of health insurance (Ref: BMIURR)**																
BMIUE	0.981	0.743	0.872	1.102	0.845	0.145	0.673	1.060	**1.761**	**< 0.001**	**1.348**	**2.301**	**1.834**	**0.004**	**1.218**	**2.760**
Free medical care	0.823	0.495	0.471	1.439	0.408	0.084	0.147	1.128	–	–	–	–	0.299	0.251	0.038	2.349
**Local fund (Ref: No)**																
Yes	**1.239**	**< 0.001**	**1.118**	**1.372**	3.089	< 0.001	2.515	3.793	**2.642**	**< 0.001**	**2.108**	**3.310**	**170.967**	**< 0.001**	**95.923**	**304.722**
**Gender (Ref: Female)**																
Male	**0.633**	**< 0.001**	**0.585**	**0.684**	0.959	0.611	0.818	1.126	1.034	0.732	0.853	1.255	1.052	0.723	0.794	1.394
**Age (Ref: 15–29 years)**																
30–44	**0.488**	**< 0.001**	**0.440**	**0.541**	0.838	0.078	0.688	1.020	1.155	0.183	0.934	1.429	**1.649**	**0.006**	**1.152**	**2.361**
45–59	**0.439**	**< 0.001**	**0.387**	**0.498**	0.858	0.226	0.670	1.099	**1.787**	**< 0.001**	**1.333**	**2.396**	**1.786**	**0.011**	**1.145**	**2.786**
60 and above	**0.641**	**< 0.001**	**0.545**	**0.754**	1.089	0.593	0.797	1.487	**2.528**	**< 0.001**	**1.696**	**3.767**	**2.708**	**< 0.001**	**1.578**	**4.648**
**Educational attainment (Ref: Primary school or below)**																
Junior high	**1.190**	**0.001**	**1.073**	**1.320**	1.171	0.109	0.966	1.420	1.159	0.232	0.910	1.475	0.937	0.708	0.665	1.319
≥Senior high	**1.376**	**< 0.001**	**1.222**	**1.549**	1.429	0.002	1.145	1.784	**1.409**	**0.012**	**1.079**	**1.839**	1.002	>0.99	0.682	1.471
**Marital status (Ref: Single)**																
Married/cohabiting	**6.062**	**< 0.001**	**5.067**	**7.253**	1.240	0.259	0.853	1.803	1.193	0.430	0.769	1.852	0.538	0.125	0.244	1.188
Divorced/widowed	**4.375**	**< 0.001**	**3.367**	**5.685**	1.163	0.571	0.689	1.962	1.412	0.316	0.719	2.772	**0.287**	**0.017**	**0.102**	**0.803**
**Monthly per capita household income ranking (Ref: Lowest)**																
Lower	0.926	0.155	0.834	1.029	0.861	0.136	0.706	1.048	1.000	>0.99	0.792	1.262	1.172	0.379	0.823	1.668
Middle	0.913	0.109	0.816	1.021	0.874	0.212	0.708	1.080	1.258	0.076	0.976	1.620	0.759	0.151	0.520	1.106
Higher	0.935	0.268	0.829	1.053	1.008	0.944	0.803	1.266	1.000	>0.99	0.769	1.300	1.120	0.569	0.758	1.654
Highest	0.946	0.410	0.830	1.079	0.950	0.684	0.740	1.218	1.029	0.845	0.775	1.366	0.747	0.205	0.476	1.173
**Self-rating of health (Ref: Poor)**																
General	**0.500**	**< 0.001**	**0.444**	**0.564**	1.350	0.006	1.091	1.671	0.833	0.223	0.620	1.118	1.068	0.724	0.740	1.542
Good	**0.593**	**< 0.001**	**0.525**	**0.670**	1.739	< 0.001	1.393	2.171	**0.627**	**0.002**	**0.467**	**0.843**	1.156	0.473	0.778	1.717
**Distance of migration (Ref: Inter-county)**																
Inter-city	**0.896**	**0.034**	**0.810**	**0.992**	0.749	0.004	0.618	0.910	**0.688**	**0.001**	**0.548**	**0.862**	**0.285**	**< 0.001**	**0.215**	**0.377**
Inter-province	**0.701**	**< 0.001**	**0.630**	**0.779**	0.618	< 0.001	0.504	0.758	**0.433**	**< 0.001**	**0.342**	**0.550**	**0.150**	**< 0.001**	**0.103**	**0.218**
**Years of residing in migration destination (Ref:** ** < 1)**																
1–4	0.948	0.382	0.841	1.068	2.303	< 0.001	1.870	2.836	0.978	0.872	0.742	1.287	0.950	0.812	0.622	1.451
5–9	0.897	0.097	0.789	1.020	2.604	< 0.001	2.066	3.281	1.137	0.401	0.843	1.533	0.948	0.817	0.605	1.485
10 and above	0.916	0.195	0.801	1.046	2.420	< 0.001	1.900	3.084	1.025	0.880	0.745	1.411	0.777	0.300	0.481	1.253
**Migration destination (Ref: Pearl River Delta)**																
Yangtze River delta	1.180	0.062	0.992	1.403	0.923	0.642	0.657	1.296	**0.652**	**0.037**	**0.437**	**0.974**	0.919	0.844	0.394	2.140
Circum-Bohai Sea	1.189	0.060	0.993	1.424	1.438	0.052	0.997	2.073	**0.500**	**0.001**	**0.333**	**0.750**	1.534	0.293	0.691	3.407
Others	1.437	< 0.001	1.228	1.682	1.051	0.755	0.768	1.438	0.927	0.685	0.644	1.335	1.937	0.076	0.933	4.022
***R* ^2^**	0.0665				0.0723				0.1062				0.4462			

BMIURR, Basic Medical Insurance for Urban and Rural Residents; BMIUE, Basic Medical Insurance for Urban Employees. Bold figures indicate results with statistical significance.

Higher odds of insurance subsidies were associated with older age (AOR = 1.787–2.528, 95% CI = 1.333–3.767, *p* < 0.001), and higher educational attainment (AOR = 1.409 for senior higher or above relative to primary, 95% CI = 1.079–1.839, *p* < 0.05). Whereas, lower odds of insurance subsidies were associated with good self-rated health (AOR = 0.627 relative to poor health, 95% CI = 0.467–0.843, *p* < 0.01), and longer distance of migration (AOR = 0.433–0.688, 95% CI = 0.342–0.862, *p* < 0.01). Those who had a local fund (AOR = 2.642, 95% CI = 2.108–3.310, *p* < 0.001) and enrolled in BMIUE (AOR = 1.761 relative to BMIURR, 95% CI = 1.348–2.301, *p* < 0.001) had higher odds of obtaining insurance subsidies (Table 4). Similar results were found in the subgroup analyses of study participants divided by household income (in quintiles), although BMIUE (relative to BMIURR) was only a significant predictor in those with lower or middle household income (Supplementary Table S4c).

Higher odds of (residential) local settlement on insurance claims were associated with older age (AOR = 1.649–2.708, 95% CI = 1.145-4.648, *p* < 0.05). Whereas, lower odds of local settlement on insurance claims were found in those who were divorced or widowed (AOR = 0.287 relative to single, 95% CI = 0.102–0.803, *p* < 0.05) and migrated across cities (AOR = 0.285 relative to cross-county, 95% CI = 0.215–0.377, *p* < 0.001) or across provinces (AOR = 0.150 relative to cross-county, 95% CI = 0.103–0.218, *p* < 0.001). Those who had a local fund (AOR = 170.967, 95% CI = 95.923–304.722, *p* < 0.001) and enrolled in BMIUE (AOR = 1.834 relative to BMIURR, 95% CI = 1.218–2.760, *p* < 0.01) had higher odds of settling insurance claims locally (Table 4). Similar results were found in the subgroup analyses of study participants divided by household income (in quintiles), although BMIUE (relative to BMIURR) was only a significant predictor in those with lower household income (Supplementary Table S4d).

Similar results were found in the logit modeling on hospitalization and use of health insurance for hospital care in the BMIURR enrollees (Supplementary Table S5) and the BMIUE enrollees (Supplementary Table S6).

### Predictors of hospital expenditure

The Heckman two-step models revealed that BMIUE was associated with a 12.7% increase in total hospital expenditure (*p* < 0.05), a 14.7% increase in reimbursement ratio (*p* < 0.001), and a 17.1% decrease in out-of-pocket payments (*p* < 0.01) in comparison with BMIURR. Local insurance funds were associated with an 18.3% decrease in out-of-pocket payments (*p* < 0.05), despite insignificant differences in total hospital expenditure (*p* = 0.124) and reimbursement ratio (*p* = 0.121) in comparison without local insurance funds. Local settlement of insurance claims was associated with a 12.6% decrease in total hospital expenditure (*p* < 0.05), a 6.6% increase in reimbursement ratio (*p* < 0.001), and a 26.2% decrease in out-of-pocket payments (*p* < 0.001) in comparison without local settlement of insurance claims (Table 5). The subgroup analyses by household income (in quintiles) showed that BMIUE was associated with increased total hospital expenditure in those with the lower household income, increased reimbursement ratio, and decreased out-of-pocket payments in those with the lowest or the highest household income. Local funds were associated with decreased out-of-pocket payments in those with the middle household income. Local settlement of insurance claims was associated with decreased total hospital expenditure in those with the middle household income, increased reimbursement ratio, and decreased out-of-pocket payments in those with the lowest, the middle, and the highest household income (Supplementary Tables S7A–C).

**Table 5:** The effect of health insurance: results of Heckman two-step model.

**Variable**	**Natural logarithm of total hospital expenditure**				**Natural logarithm of out-of-pocket payments**				**Reimbursement ratio of hospital expenditure**			
	**Selection model (hospital admission**, ***n*** = **13,065)**		**Outcome model (*****n*** = **2,186)**		**Selection model (hospital admission**, ***n*** = **13,065)**		**Outcome model (*****n*** = **2,186)**		**Selection model (insurance reimbursement**, ***n*** = **3,186)**		**Outcome model (*****n*** = **2,229)**	
	**Coef**	***P***	**Coef**	***P***	**Coef**	***P***	**Coef**	***P***	**Coef**	***P***	**Coef**	***P***
**Type of health insurance (Ref: BMIURR)**												
BMIUE	0.020	0.625	**0.127**	**0.011**	0.020	0.625	**-0.171**	**0.005**	0.320	< 0.001	**0.147**	**< 0.001**
Free medical care	−0.133	0.511	0.504	0.075	−0.133	0.511	0.064	0.853	5.954	>0.99	**0.290**	**< 0.001**
**Local fund (Ref: no)**												
Yes	0.439	< 0.001	−0.096	0.124	0.439	< 0.001	–**0.183**	**0.017**	0.574	< 0.001	0.047	0.121
**Location of insurance settlement (Ref: Non-residential location)**												
Residential location			**0.126**	**0.012**			**0.262**	**< 0.001**			**0.066**	**< 0.001**
**Gender (Ref: Female)**												
Male	−0.268	< 0.001	**0.145**	**0.007**	−0.268	< 0.001	0.065	0.324	0.025	0.670	**0.031**	**0.004**
**Age (Ref: 15–29 years)**												
30–44	−0.386	< 0.001	0.018	0.784	−0.386	< 0.001	−0.075	0.346	0.090	0.164	**0.033**	**0.017**
45–59	−0.358	< 0.001	0.100	0.185	−0.358	< 0.001	−0.045	0.624	0.356	< 0.001	**0.052**	**0.039**
60 and above	−0.052	0.392	0.053	0.498	−0.052	0.392	−0.120	0.211	0.549	< 0.001	**0.066**	**0.050**
**Educational attainment (Ref: Primary school or below)**												
Junior high	0.139	< 0.001	**0.166**	**0.004**	0.139	< 0.001	**0.195**	**0.005**	0.094	0.202	−0.021	0.136
≥Senior high	0.270	< 0.001	**0.184**	**0.007**	0.270	< 0.001	**0.221**	**0.008**	0.210	0.009	−0.019	0.282
**Marital status (Ref: Single)**												
Married/ Cohabiting	1.010	< 0.001	0.184	0.253	1.010	< 0.001	0.359	0.068	0.116	0.386	–**0.062**	**0.021**
Divorced/ Widowed	0.823	< 0.001	−0.084	0.628	0.823	< 0.001	0.010	0.961	0.250	0.220	−0.025	0.503
**Monthly per capita household income ranking (Ref: Lowest)**												
Lower	−0.045	0.262	0.062	0.252	−0.045	0.262	0.041	0.539	0.002	0.982	0.002	0.912
Middle	−0.010	0.814	0.066	0.239	−0.010	0.814	0.031	0.653	0.137	0.073	0.008	0.600
Higher	−0.019	0.678	**0.120**	**0.040**	−0.019	0.678	0.127	0.075	0.011	0.890	−0.002	0.918
Highest	−0.015	0.760	**0.156**	**0.013**	−0.015	0.760	0.131	0.091	0.025	0.773	−0.002	0.919
**Self-rated health (Ref: Poor)**												
General	−0.341	< 0.001	–**0.283**	**< 0.001**	−0.341	< 0.001	–**0.300**	**0.001**	−0.111	0.204	0.005	0.759
Good	−0.261	< 0.001	–**0.484**	**< 0.001**	−0.261	< 0.001	–**0.505**	**< 0.001**	−0.281	0.001	0.006	0.789
**Distance of migration (Ref: Inter-county)**												
Inter-city	−0.175	< 0.001			−0.175	< 0.001			−0.219	0.001	−0.009	0.562
Inter-province	−0.428	< 0.001			−0.428	< 0.001			−0.484	< 0.001	−0.016	0.552
**Years of residing in migration destination (Ref:** ** < 1)**												
1–4	0.102	0.035			0.102	0.035			−0.010	0.909		
5–9	0.102	0.047			0.102	0.047			0.078	0.394		
10 and above	0.102	0.055			0.102	0.055			0.015	0.880		
**Migration destination (Ref: Pearl River Delta)**												
Yangtze River delta	0.002	0.980	**0.227**	**0.016**	0.002	0.980	**0.520**	**< 0.001**	−0.256	0.037	–**0.073**	**0.007**
Circum-Bohai Sea	0.065	0.332	**0.195**	**0.039**	0.065	0.332	**0.465**	**< 0.001**	−0.421	0.001	–**0.082**	**0.010**
Others	0.201	0.001	−0.041	0.652	0.201	0.001	**0.238**	**0.032**	−0.046	0.676	–**0.077**	**< 0.001**
**Mills**	**Coef**		***P***		**Coef**		***P***		**Coef**		***P***	
**λ**_**i**_	0.095		0.544		0.161		0.400		−0.009		0.937	

BMIURR, Basic Medical Insurance for Urban and Rural Residents; BMIUE, Basic Medical Insurance for Urban Employee. Bold figures indicate results with statistical significance.

Local funds and local settlement of insurance claims appeared to make a significant difference for the BMIURR enrollees (Supplementary Table S8), but not for the BMIUE enrollees (Supplementary Table S9).

Although both “local fund” and “location of insurance settlement” were significant predictors (*p* < 0.05) of total hospital expenditure, out-of-pocket payments, and reimbursement ratio of hospital expenditure when they were entered into the models separately, the effect of “local fund” became insignificant for total hospital expenditure and reimbursement ratio (*p* > 0.05) when both variables were entered into the models simultaneously (Supplementary Tables S10A–C).

## Discussion

### Principal findings

In this study, we found that the use of health insurance for hospital care is inadequate for internal migrants in mainland China. Overall, only about two-thirds (69.96%) of the hospitalized migrants were subsidized by insurance funds. The existence of multiple funds linked to the Hukou location has created great geographic and administrative barriers to fully realizing the benefits of the social health insurance programs. There exist significant inequalities in health insurance usage for hospital care in internal migrants in mainland China. Insurance benefits for hospital care vary by insurance funds, fund location, and where insurance claims are settled. BMIUE, local funds, and local settlement of insurance claims are associated with higher levels of health insurance usage and lower out-of-pocket payments. Longer distance of migration is a significant predictor of a lower likelihood of insurance subsidy and local settlement of insurance claims. The availability of local funds is beneficial to BMIURR enrollees, but not so much for BMIUE enrollees.

### Comparisons and possible explanations

The level of use of health insurance, especially BMIURR, for hospital care was low in the insured internal migrants who reported illness or injury over the past year, despite a high hospitalization rate (28.9%). We found that <70% of the hospitalized migrants (64.37% of BMIURR enrollees) were subsidized by insurance funds, compared with an overall of 90% across all patient populations in the same year in mainland China (48). Meanwhile, only 59% of total hospital expenditure in BMIUE enrollees and 42% in BMIURR enrollees were paid by insurance funds, compared with an overall of 71.8% across all patient populations with BMIUE and 56.1% across all patient populations with BMIURR in the same year in mainland China (49). These findings provide evidence support to the concern of the government about the financial and administrative barriers for internal migrants to enjoy the benefits of social health insurance (50).

The design of the social health insurance programs presents a significant barrier for internal migrants to fully enjoy the insurance benefits. We found that the vast majority (75.51%) of internal migrants enrolled in BMIURR, and BMIURR (relative to BMIUE) is a significant predictor of lower odds of insurance subsidy, lower insurance reimbursement ratio, and higher out-of-pocket payments, despite lower total hospital expenditure. These results are consistent with the findings of previous studies (8, 19, 35–37). Compared with BMIUE, BMIURR usually has a smaller funding pool, resulting in a lower reimbursement ratio and lower use for hospital care. Although BMIURR funds cover a large number of internal migrants, the portability of fund benefits is low, which could lead to low use of insurance funds as revealed in our study and others (8, 19, 35–37).

Fund location is another significant determinant of insurance benefits. Our study showed that most (>70%) internal migrants had a fund outside of their residential location, and having a local fund is a significant predictor of health insurance usage for hospital care. Local fund is associated with higher odds of insurance subsidy, higher insurance reimbursement ratio, and lower out-of-pocket payments for BMIURR enrollees. These results are consistent with the findings of previous studies (16, 28, 51, 52). The location of funds has limited effects on BMIUE enrollees, but it makes a big difference in easing the financial burden of individual patients enrolled with BMIURR. It is important to note that there may be additional indirect costs (such as travel and loss of work income) associated with the use of non-local funds (53, 54). BMIURR enrollees without a local fund have to balance the needs of healthcare and insurance entitlements. Insurance funds often impose stricter conditions on the use of insurance for hospital services outside of the fund location. For example, the expenses have to incur in the designated hospitals (28). Meanwhile, higher levels of deductibles and co-payment requirements are set up to discourage the use of insurance funds for hospital care outside of the fund location (42, 54). Previous studies have shown that many internal migrants could not get their hospital expenditure reimbursed because their hospital care was not covered by their insurance funds; it was too inconvenient to travel to settle insurance claims; and the reimbursement procedure was too complex to navigate (31, 55–58). However, traveling to the fund location for hospital care is not necessarily a cheaper option. A previous study estimated that travel costs and income loss account for 27–35% of the total costs of internal migrants in China who sought hospital care outside of their residential location (56).

The Chinese government initiated the on-the-spot settlement of medical bills to address the above-mentioned dilemma (59–61). However, the policy mainly targets relocated retirees and migrated long-term residents. BMIUE enrollees usually have higher access to on-the-spot settlement facilities than BMIURR enrollees, although BMIURR enrollees are those most in need of such facilities as indicated by the findings of our study. We found that local settlement on insurance claims is associated with lower hospital expenditure and lower out-of-pocket payments of the hospitalized BMIURR enrollees. These results are consistent with the findings of previous studies (17). On-the-spot settlement of hospital bills also reduces the deposit payments required, simplifies the procedure of insurance claims, and minimizes travel costs (62). However, the vast majority of internal migrants are covered by BMIURR and have difficulties accessing the already limited on-the-spot settlement facilities. By the end of June 2018, a total of 10,015 medical institutions participated in the cross-provincial on-the-spot insurance settlement program in China, with an average of only 2,491 claims settled on-the-spot per day (63). The lack of interconnections among the provincial information platforms adds an additional layer of obstacles to the on-the-spot settlement initiative (64).

We found that age, gender, educational attainment, income, self-rated health, and marital status are significant predictors of health insurance usage for hospital care. Older age is associated with a higher likelihood of insurance subsidy and local settlement of insurance claims, and a higher reimbursement ratio from insurance funds. Male gender is associated with higher total hospital expenditure and higher insurance reimbursement ratio. Higher levels of educational attainment are associated with a higher likelihood of insurance subsidy, and higher total hospital expenditure and out-of-pocket payments. Higher income is associated with higher total hospital expenditure. Good self-rated health is associated with a lower likelihood of insurance subsidy, but lower total hospital expenditure and out-of-pocket payments. Divorce or widowhood is associated with a lower likelihood of local settlement of insurance claims, but a higher insurance reimbursement ratio. These results are consistent with the findings of previous studies (30, 31) and align well with Andersen's model.

Distance of migration is another significant predictor of health insurance usage for hospital care. We found that a longer distance of migration is associated with lower odds of insurance subsidy and lower odds of local settlement on insurance claims. A previous study found that internal migrants may forfeit insurance entitlements due to the need for long-distance travel (65). These provide additional evidence to support the importance of addressing the geographic and administrative barriers for internal migrants to enjoy health insurance benefits (66). Internal migrants, in particular those who travel long-distance, may face challenges to navigate through the local health system and policies in their migration destination.

### Strengths and weaknesses of the study

This study has several strengths. The dataset used in this study was derived from a nationally representative survey of internal migrants. This is the first study systematically analyzing the association between health insurance design and the use of health insurance for hospital care among internal migrants in mainland China.

There are several limitations in this study. It was focused on the use of social health insurance for hospital care only. Data were collected through self-report, which is subject to recall bias. The cross-sectional design of this study prevents us from drawing any causal conclusions. The analyses did not distinguish between rural-to-urban and urban-to-urban migrants, although the vast majority of internal migrants flew from rural to urban. Apart from self-rated health, data measuring illness severity were not available. Further studies should expand the scope of outcome indicators and examine fund transaction records.

## Conclusion

Health insurance usage for hospital care for internal migrants in mainland China is inadequate, especially for those enrolled in BMIURR. The design of the current social insurance health programs cannot meet the needs of internal migrants. The existence of multiple funds linked to the Hukou location has created great geographic and administrative barriers to fully realizing the benefits of the social health insurance programs. The governmental initiative to allow the transfer of insurance funds and settle insurance claims on-the-spot represents a promising effort. However, the intended outcomes of such an effort can be jeopardized by the low level of the funding pool and the need to contain insurance costs. Meanwhile, there exist significant inequalities in health insurance usage for hospital care in internal migrants in mainland China across and within insurance funds. The gap between BMIUE and BMIURR remains to be a great concern. Increasing policy attention need to be paid to fund location and costs of claim settlement, in particular for those enrolled in BMIURR.

To improve health insurance usage for hospital care, internal migrants need to be empowered. The complicated healthcare and health insurance systems are very difficult to navigate. Adding to the complexity are the variations in insurance policies across regions and across funds. Higher levels of awareness of insurance fund transferability and on-the-spot settlement systems for hospital bills need to be ensured.

Although the fundamental solution for an effective and equitable insurance system rests on expanded funding pools (at higher levels) and better integration of insurance policies, governments, employers, and consumers all play an important role in improving the current system. The BMIURR programs should be prioritized for fund portability and on-the-spot claim settlement initiatives. More health facilities should be encouraged to participate in the on-the-spot settlement program through policy incentives supported by interconnected information platforms.

## Data availability statement

The datasets used in this study are publicly available and can be accessed *via* Migrant Population Service Center, National Health Commission of China. Requests to access these datasets should be directed to https://www.chinaldrk.org.cn/wjw/#/home.

## Ethics statement

As this study was a secondary analysis of de-identified data collected by the government, ethics approval has been exempted. The 2018 China Migrants Dynamic Survey was approved by the China National Bureau of Statistics (No. Guotongzhi [2018] No. 45), and written informed consent was obtained from all participants at the time of data collection. The use of the data for this study was approved by the Migrant Population Service Centre, National Health Commission of China. All procedures performed in this study were in accordance with the 1964 Helsinki declaration and its later amendments or comparable ethical standards.

## Author contributions

QY contributed to the study design, data analyses, and drafting of the manuscript. HL contributed to data analyses, data interpretation, and drafting of the manuscript. CL contributed to the interpretation of the results and writing of the manuscript. All authors have read and approved the final version of the manuscript.

## Funding

The study was funded by the National Natural Science Foundation of China (72174149 and 71603188), Humanity, and Social Science Foundation from the Ministry of Education of China (21YJAZH102), and the Key Research Institute Project of Humanity and Social Science of the Ministry of Education of China (1203-413100050).

## Conflict of interest

The authors declare that the research was conducted in the absence of any commercial or financial relationships that could be construed as a potential conflict of interest.

## Publisher's note

All claims expressed in this article are solely those of the authors and do not necessarily represent those of their affiliated organizations, or those of the publisher, the editors and the reviewers. Any product that may be evaluated in this article, or claim that may be made by its manufacturer, is not guaranteed or endorsed by the publisher.

## References

[1] He X Shi S. The impact of population mobility on regional economic growth: an empirical analysis based on panel data of prefecture-level cities in China. J Finan Econ. (2021) 3:63–70. 10.19622/j.cnki.cn36-1005/f.2021.03.008

[2] Shi G Li Z. Research on the mechanism of population mobility promoting regional economic growth: based on panel data of the Yangtze River delta urban agglomeration. East China Econ Manag. (2020) 34:10–8. 10.19629/j.cnki.34-1014/f.191125008

[3] Zhang Z Yang S. A review on the concepts, data and fields of floating population research. Chin J Popul Sci. (2013) 6:102–12.

[4] Wang P. The Total Population Has Maintained Growth the Level of Urbanization Has Been Steadily Improved. China Economic Net (2022). Available from: http://www.ce.cn/xwzx/gnsz/gdxw/202201/18/t20220118_37264987.shtml (accessed July 24, 2022).

[5] Wang J Zheng J Wang P Qi L. Migration and health in China: bridging the gaps in policy aims and the reality of medical services to migrants. J Public Administr. (2014) 9:29–45. 10.3969/j.issn.1674-2486.2014.04.002

[6] Website CNS. China's Floating Population Exceeds 240 Million How Access to Health Services Improves English Translation. (2019). Available from: https://baijiahao.baidu.com/s?id=1652269305128071421&wfr=spider&for=pc (accessed July 24, 2022).

[7] Zhang X. A study on the heterogeneity of multidimensional poverty in difficult families. Stat Decis. (2021) 37:78–81. 10.13546/j.cnki.tjyjc.2021.04.017

[8] Fan X. The health conditions and problems of the migrant population and countermeasures. Macroecon Manag. (2019) 42–7. 10.19709/j.cnki.11-3199/f.2019.04.010

[9] Yang J. Double-dual property of Hukou system and social integration of internal migrants in the context of new-blueprint urbanization in China. J Renmin Univ China. (2017) 31:119–28. 10.3969/j.issn.1000-5420.2017.04.013

[10] Niu J Zheng Z Zhang L Zeng X. Labor migrants' working and living environments and the related health impacts—Evidences from Shenzhen. Popul Res. (2011) 35:64–75.

[11] Baker DW Sudano JJ Albert JM Borawski EA Dor A. Lack of health insurance and decline in overall health in late middle age. N Engl J Med. (2001) 345:2887. 10.1056/NEJMsa002887 11596591

[12] Arrow KJ. Uncertainty and the welfare economics of medical care. Bull World Health Organ. (2004) 82:141–9. 15042238 PMC2585909

[13] Cai X Yang F Bian Y. Gap analysis on hospitalized health service utilization in floating population covered by different medical insurances ——Case Study from Jiangsu Province, China. Int J Equity Health. (2019) 18:84. 10.1186/s12939-019-0992-4 31182101 PMC6558691

[14] Pan L Wang C Cao X Zhu H Luo L. Unmet healthcare needs and their determining factors among unwell migrants: a comparative study in Shanghai. Int J Environ Res Public Health. (2022) 19:5499. 10.3390/ijerph19095499 35564894 PMC9103782

[15] Feng G Qian N Qu C Zhang Y. The impact of medical insurance models on the accessibility of medical and health services among the migrating population. J Dalian Univ Technol. (2017) 38:144–50. 10.19525/j.issn1008-407x.2017.01.022

[16] Meng Y Han J. The impact of medical insurance on the health service utilization of the floating population: empirical analysis based on the 2017 China migrants dynamic survey. Chin J Popul Sci. (2019) 5:110.

[17] Zhang X Zhang L. The impact of instant reimbursement of cross-regional medical services on hospitalization costs incurred by the floating population-evidence from China. Healthcare. (2022) 10:1099. 10.3390/healthcare10061099 35742150 PMC9223039

[18] Chen S Zhang Q Yang D Wang J Chen M. Analysis on health service utilization and influencing factors of the young migrants in Guangdong Province. Chinese Health Econ. (2020) 39:77–81. 10.7664/CHE20200918

[19] Zhang F Shi X Zhou Y. The impact of health insurance on healthcare utilization by migrant workers in China. Int J Environ Res Public Health. (2020) 17:1852. 10.3390/ijerph17061852 32178431 PMC7143864

[20] Lu X. Studying on the influence of medical insurance on health service utilization of floating elderly people based on counterfactual estimation of propensity score matching method. Chin Health Serv Manag. (2019) 36:657–60.

[21] Tang D Wang J. Basic public health service utilization by internal older adult migrants in China. Int J Environ Res Public Health. (2021) 18:270. 10.3390/ijerph18010270 33401371 PMC7795646

[22] Wang X Jianbo L Yang Y. Status and influencing factors of receiving health education and personal health record establishment among elderly migrant population in China. Chinese J Public Health. (2021) 37:203–8. 10.11847/zgggws1128961

[23] Yan Q Tong L. Utilization of basic public health services and its influence factors among young migrants. Chinese J Public Health. (2019) 35:680–4. 10.11847/zgggws1119401

[24] Ward L Franks P. Changes in health care expenditure associated with gaining or losing health insurance. Ann Intern Med. (2007) 146:768–74. 10.7326/0003-4819-146-11-200706050-00005 17548408

[25] Wagstaff A Lindelow M. Can insurance increase financial risk? The Curious Case of Health Insurance in China. J Health Econ. (2008) 27:990–1005. 10.1016/j.jhealeco.2008.02.002 18342963

[26] Zhou L Zhu Z. A Study on the decision of medical insurance on the part of floating population and its impact on medical expenditures. J Nanjing Audit Univ. (2017) 4:66–75.

[27] Wang C. The Effective coverage rate and distribution character-istics of China's basic medical insurance: analysis based on multi-source data. Chinese Soc Sec Rev. (2020) 4:67–84.

[28] Meng Y Zhang X Wang J. “Lock-in” and “Pull-Back”: the lmpact of medical lnsurance on the residence lntention of floating population. J Northeast Univ. (2021) 23:67–75. 10.15936/j.cnki.1008-3758.2021.04.009

[29] Ma C Zhang Y Li Y Wang Y Jiang Y Wang X. Healthcare, insurance, and medical expenditure of the floating population in Beijing, China. Front Public Health. (2020) 8:375. 10.3389/fpubh.2020.00375 32850597 PMC7423999

[30] Guo J Zhou Q Weng H Wu Y. Analysis on multilevel logistic regression model of the utilization of health services for migrant population and influencing factors. Chinese Health Econ. (2015) 34:50–2. 10.7664/CHE20150314

[31] Yin Q Xu Q Zheng Y. Status and influencing factors of hospitalization service utilization among migrant population. Chinese J Public Health. (2017) 33:448–51. 10.11847/zgggws2017-33-03-2827775826

[32] Zhou Q Liu G. The difference of benefits from health insurance: based on the study of the local population and migrants. Nankai Econ Stud. (2016) 1:77–94. 10.14116/j.nkes.2016.01.005

[33] Gao J Chu D Ye T Gong D. Empirical analysis of beneficial equality of the basic medical insurance for migrants in China. Discrete Dyn Nat Soc. (2021) 2021:1–11. 10.1155/2021/7708605

[34] Hao Y Zhou J Zhang J. Public health insurance, urbanization and rural-to-urban migrants' consumption. Comp Econ Soc Syst. (2022) 1:91–104. 10.3969/j.issn.1003-3947.2022.01.010

[35] Zhou Y Huang Y. Can the basic medical insurance system improve the relative inequality of migrant workers? Finan Econ. (2021) 84–97. 10.3969/j.issn.1000-8306.2021.10.008

[36] Jiang H. Medical insurance and medical service utilization of floating population—Based on dynamic monitoring data of national floating population. World Surv Res. (2016) 7:14–20. 10.13778/j.cnki.11-3705/c.2016.07.003

[37] Li J Huang Y Nicholas S Wang J. China's new cooperative medical scheme's impact on the medical expenses of elderly rural migrants. Int J Environ Res Public Health. (2019) 16:4953. 10.3390/ijerph16244953 31817627 PMC6950318

[38] Migrant Population Service Center. The 2018 China Migrants Dynamic Survey Technical Document. National Health Commission P.R.China (2018). Available from: https://www.chinaldrk.org.cn/wjw/#/home (accessed July 24, 2022).

[39] Li Y Lu S. A Review of the development of the Anderson model's theoretical construction and analysis path. Chinese Health Serv Manag. (2017) 34:324–7+334.

[40] Li Y Lu S. The development, application and implications of the Anderson model in the field of healthcare. Chinese J Health Policy. (2017) 10:77–82. 10.3969/j.issn.1674-2982.2017.11.013

[41] Yao Q Liu C Sun J. Inequality in health services for internal migrants in China: A National Cross-Sectional Study on the Role of Fund Location of Social Health Insurance. Int J Environ Res Public Health. (2020) 17:6327. 10.3390/ijerph17176327 32878066 PMC7504160

[42] People's Government of Anhui Provincial. Policy Interpretation of Anhui Province Unified Basic Medical Insurance and Serious Disease Insurance Guarantee Treatment Plan (Trial). (2019). Available from: https://www.ah.gov.cn/public/1681/7977961.html?ivk_sa=1024320u (accessed July 24, 2022).

[43] Zhang L. The strategy and path of the “instant reimbursement of cross-provincial outpatient expenses”: taking “Yangtze River Delta” region as an example. Nanjing J Soc Sci. (2021) 6:70–7. 10.15937/j.cnki.issn1001-8263.2021.06.009

[44] Zhu M Shi X. The impact of medical insurance coverage on migrant population's catastrophic health expenditure. Chin J Popul Sci. (2016) 6:47–57.

[45] Heckman JJ. Sample selection bias as a specification error. Econometrica. (1979) 47:153–61. 10.2307/1912352

[46] Tan Y Lu Q. Risk Aversion, social learning and farmer's adoption of modern irrigation technology: an empirical analysis by heckman sample selection model. Resourc Environ Yangtze Basin. (2021) 30:234–45. 10.11870/cjlyzyyhj202101022

[47] Vatcheva KP Lee M McCormick JB Rahbar MH. Multicollinearity in regression analyses conducted in epidemiologic studies. Epidemiology. (2016) 6:227. 10.4172/2161-1165.1000227 27274911 PMC4888898

[48] National Center for Health Statistics. The Report of National Health Service Survey in China, 2018. Beijing: People's Medical Publishing House (2021).

[49] National Healthcare Security Administration. Statistical Bulletin on the Development of National Basic Medical Insurance. (2018). Available from: http://www.nhsa.gov.cn/art/2019/6/30/art_7_1477.html (accessed July 24, 2022).

[50] Qin X Pan J Liu GG. Does participating in health insurance benefit the migrant workers in China? An empirical investigation China. Econ Rev. (2014) 30:263–78. 10.1016/j.chieco.2014.07.009

[51] Yao Q Chen A. Influencing paths of medical insurance enrollment location on the health conditions of elderly migrants in China: based on data from 2015 China migrants dynamic survey. Chinese J Health Policy. (2022) 15:57–63. 10.3969/j.issn.1674-2982.2022.01.008

[52] Bai L Gu H A. Study on the influence of the difference of medical insurance participating areas on the healthcare utilization of the elderly floating population under the background of medical settlement in different places. Lanzhou Acad J. (2021) 5:181–96. 10.3969/j.issn.1005-3492.2021.05.013

[53] Zhong H. Effect of patient reimbursement method on health-care utilization: evidence from China. J Health Econ. (2011) 20:1312–29. 10.1002/hec.1670 20882574

[54] Qiu F Liu J Zhan HJ. Migration and health—Freedom of movement and social benefits for Chinese migrant workers. Sustainabilit.y. (2021) 13:12371. 10.3390/su13221237112280381

[55] Guo L Bao Y Liu X Nie H Sun W Dai H. Analysis of quality of utilizing basic medical services by floating population in Shanghai. J Shanghai Jiaotong Univ. (2016) 36:105–9. 10.3969/j.issn.1674-8115.2016.01.021

[56] Tang Z. Intergenerational comparison of migrant workers' choice of public medical services—Based on the data of the 2014 National Floating Population Dynamic Monitoring Survey in Jiangsu, Shanghai and Zhejiang Provinces. Jiangsu Soc Sci. (2018) 3:117–23. 10.13858/j.cnki.cn32-1312/c.2018.03.016

[57] Tang Z. Intergenerational comparison of migrant workers' participation in medical insurance and their choice of medical treatment behavior——Based on the Data of the 2014 National Floating Population Health and Family Planning Dynamic Monitoring Survey in Nine Eastern Provinces and Cities. Soc Sci Guangdong. (2018) 1:210–7. 10.3969/j.issn.1000-114X.2018.01.023

[58] Qian Z Lin S Hou Z. Significantly improving local hospitalization rate of migrants under urban basic medical insurance: based on the evidence of national migrant dynamic supervision survey 2014. Chinese Health Econ. (2016) 35:44–6. 10.7664/CHE20160912

[59] National Healthcare Security Administration. Notice on Further Carry out on-the-Spot Settlements of Medical Bills for across-Provincial Medical Treatment. (2022). Available from: http://www.nhsa.gov.cn/art/2022/7/26/art_104_8629.html (accessed July 24, 2022).

[60] Ministry of Human Resources Social Security of the People's Republic of China. Notice on Enabling on-the-Spot Settlements of Medical Bills for across-Provincial Medical Treatment. (2016). Available from: http://www.mohrss.gov.cn/SYrlzyhshbzb/shehuibaozhang/zcwj/201612/t20161215_262040.html (accessed July 24, 2022).

[61] Ministry of Human Resources Social Security of the People's Republic of China. Notice on Carry out on-the-Spot Settlements of Medical Bills for across-Provincial Medical Treatment. (2017). Available from: http://www.mohrss.gov.cn/SYrlzyhshbzb/shehuibaozhang/zcwj/yiliao/201801/t20180108_286172.html (accessed July 24, 2022).

[62] Zhen C Wei J Wan H Zhao G. Reengineering and practice of direct settlement process of nonlocal hospitalization bills. Chinese Hosp Manag. (2019) 39:56–8.

[63] National Healthcare Security Administration. Public Service Information Release of Basic Medical Insurance for Cross-Provincial Medical Treatment in Different Places (the First Issue). (2018). Available from: http://www.nhsa.gov.cn/art/2018/8/15/art_114_7166.html (accessed July 24, 2022).

[64] Pan Y Chen S Chen M Zhang P Long Q Xiang L. Disparity in reimbursement for tuberculosis care among different health insurance schemes: evidence from three counties in central China. Infect Dis Poverty. (2016) 5:46–54. 10.1186/s40249-016-0102-4 26812914 PMC4729161

[65] Wang H Liang D Zhang D Hou Z. How does domestic migration pose a challenge in achieving equitable social health insurance benefits in China? A National Cross-Sectional Study. BMJ Open. (2022) 12:e060551. 10.1136/bmjopen-2021-060551 35998949 PMC9403113

[66] Li H Yang J. Regional disparities of medical insurance of urban employees of migrants. Popul Soc. (2017) 33:3–12. 10.14132/j.2095-7963.2017.03.001

