# Supplementary material for: Use of social health insurance for hospital care by internal migrants in China—Evidence from the 2018 China migrants dynamic survey
Source: Front Public Health. 2022 Nov 24;10:1008720. doi: 10.3389/fpubh.2022.1008720 (PMC9729771; doi:10.3389/fpubh.2022.1008720)
Supplement: Supplementary file 1 [file Data_Sheet_1.docx]

# Table S1. Multicollinearity tests of independent variables measured by variance inflation factor (VIF)

| **Variable** | **Sample for modelling of hospital expenditure (n=2186)** | | **Sample for modelling of reimbursement ratio (n=2229)** | |
| --- | --- | --- | --- | --- |
|  | **VIF** | **Tolerance (1/VIF)** | **VIF** | **Tolerance (1/VIF)** |
| **Type of health insurance** (Ref: BMIURR) | | | | |
| BMIUE | 1.68 | 0.597 | 1.72 | 0.582 |
| Free medical care | 1.03 | 0.971 | 1.03 | 0.968 |
| **Local fund** (Ref: no) | | | | |
| yes | 1.96 | 0.510 | 2.20 | 0.454 |
| **Location of insurance settlement** (Ref: None-residential location) | | | | |
| Residential location | 1.66 | 0.601 | 1.81 | 0.553 |
| **Gender** (Ref: Female) | | | | |
| Male | 1.20 | 0.832 | 1.21 | 0.829 |
| **Age** (Ref: 15-29 years) | | | | |
| 30-44 | 1.66 | 0.601 | 1.66 | 0.601 |
| 45-59 | 2.19 | 0.456 | 2.19 | 0.456 |
| 60 and above | 2.33 | 0.430 | 2.32 | 0.431 |
| **Educational attainment** (Ref: Primary school or below) | | | | |
| Junior high | 2.00 | 0.500 | 2.01 | 0.498 |
| ≥Senior high | 2.68 | 0.373 | 2.70 | 0.370 |
| **Marital status** (Ref: Single) | | | | |
| Married/ Cohabiting | 2.31 | 0.433 | 2.32 | 0.431 |
| Divorced/ Widowed | 2.35 | 0.425 | 2.36 | 0.424 |
| **Monthly per capita household income ranking** (Ref: Lowest) | | | | |
| Lower | 1.45 | 0.691 | 1.45 | 0.690 |
| Middle | 1.47 | 0.679 | 1.49 | 0.673 |
| Higher | 1.53 | 0.656 | 1.54 | 0.649 |
| Highest | 1.56 | 0.640 | 1.59 | 0.629 |
| **Self-rated health** (Ref: Poor) | | | | |
| General | 2.00 | 0.501 | 2.01 | 0.496 |
| Good | 2.91 | 0.343 | 2.94 | 0.341 |
| **Distance of migration** (Ref: Inter-county) | | | | |
| Inter-city | - | - | 1.70 | 0.588 |
| Inter-province | - | - | 1.97 | 0.507 |
| **Migration destination** (Ref: Pearl River Delta) | | | | |
| Yangtze River delta | 2.73 | 0.366 | 4.40 | 0.227 |
| Circum-Bohai Sea | 2.90 | 0.345 | 2.90 | 0.345 |
| others | 4.17 | 0.240 | 2.78 | 0.360 |
| **Mean VIF** | 2.08 |  | 2.10 |  |

# Table S2. Use of health insurance for hospital care in study participants: subgroup analyses in participants divided by per capital monthly household income (in quintiles)

| **Variable** | **Coding of value** | **With local insurance** | | **With non-local insurance** | | **Total** | | ***P*** |
| --- | --- | --- | --- | --- | --- | --- | --- | --- |
|  |  | **n** | **%** | **n** | **%** | **n** | **%** |  |
| **Participants with the lowest per capita household income (n=4300)** | | | | | | | | |
| **Hospitalisation** | 0=No | 622 | 66.45 | 2,355 | 70.01 | 2,977 | 69.23 | 0.037 |
|  | 1=Yes | 314 | 33.55 | 1,009 | 29.99 | 1,323 | 30.77 |  |
| **Among those hospitalised (n=1323)** | | | | | | | | |
| **Location of admitted hospital** | 0=None-residential location | 36 | 11.46 | 341 | 33.80 | 377 | 28.50 | <0.001 |
|  | 1=Residential location | 278 | 88.54 | 668 | 66.20 | 946 | 71.50 |  |
| **Insurance reimbursement** | 0=No | 56 | 20.44 | 240 | 36.09 | 296 | 31.52 | <0.001 |
|  | 1=Yes | 218 | 79.56 | 425 | 63.91 | 643 | 68.48 |  |
| **Among those with insurance reimbursement (n=643)** | | | | | | | | |
| **Location of insurance settlement** | 0=None-residential location | 4 | 1.83 | 243 | 57.18 | 247 | 38.41 | <0.001 |
|  | 1=Residential location | 214 | 98.17 | 182 | 42.82 | 396 | 61.59 |  |
| **Participants with the lower household income (n=3214)** | | | | | | | | |
| **Hospitalisation** | 0=No | 546 | 66.83 | 1,728 | 72.09 | 2,274 | 70.75 | 0.004 |
|  | 1=Yes | 271 | 33.17 | 669 | 27.91 | 940 | 29.25 |  |
| **Among those hospitalised (n=940)** | | | | | | | | |
| **Location of admitted hospital** | 0=None-residential location | 38 | 14.02 | 233 | 34.83 | 271 | 28.83 | <0.001 |
|  | 1=Residential location | 233 | 85.98 | 436 | 65.17 | 669 | 71.17 |  |
| **Insurance reimbursement** | 0=No | 41 | 17.83 | 181 | 41.90 | 222 | 33.53 | <0.001 |
|  | 1=Yes | 189 | 82.17 | 251 | 58.10 | 440 | 66.47 |  |
| **Among those with insurance reimbursement (n=440)** | | | | | | | | |
| **Location of insurance settlement** | 0=None-residential location | 3 | 1.59 | 146 | 58.17 | 149 | 33.86 | <0.001 |
|  | 1=Residential location | 186 | 98.41 | 105 | 41.83 | 291 | 66.14 |  |
| **Participants with the middle household income (n=2855)** | | | | | | | | |
| **Hospitalisation** | 0=No | 540 | 66.18 | 1,512 | 74.15 | 2,052 | 71.87 | <0.001 |
|  | 1=Yes | 276 | 33.82 | 527 | 25.85 | 803 | 28.13 |  |
| **Among those hospitalised (n=803)** | | | | | | | | |
| **Location of admitted hospital** | 0=None-residential location | 52 | 18.84 | 168 | 31.88 | 220 | 27.40 | <0.001 |
|  | 1=Residential location | 224 | 81.16 | 359 | 68.12 | 583 | 72.60 |  |
| **Insurance reimbursement** | 0=No | 30 | 13.51 | 127 | 36.08 | 157 | 27.35 | <0.001 |
|  | 1=Yes | 192 | 86.49 | 225 | 63.92 | 417 | 72.65 |  |
| **Among those with insurance reimbursement (n=417)** | | | | | | | | |
| **Location of insurance settlement** | 0=None-residential location | 6 | 3.13 | 147 | 65.33 | 153 | 36.69 | <0.001 |
|  | 1=Residential location | 186 | 96.88 | 78 | 34.67 | 264 | 63.31 |  |
| **Participants with the higher household income (n=2548)** | | | | | | | | |
| **Hospitalisation** | 0=No | 607 | 68.82 | 1,206 | 72.39 | 1,813 | 71.15 | 0.059 |
|  | 1=Yes | 275 | 31.18 | 460 | 27.61 | 735 | 28.85 |  |
| **Among those hospitalised (n=735)** | | | | | | | | |
| **Location of admitted hospital** | 0=None-residential location | 35 | 12.73 | 143 | 31.09 | 178 | 24.22 | <0.001 |
|  | 1=Residential location | 240 | 87.27 | 317 | 68.91 | 557 | 75.78 |  |
| **Insurance reimbursement** | 0=No | 42 | 17.50 | 109 | 35.86 | 151 | 27.76 | <0.001 |
|  | 1=Yes | 198 | 82.50 | 195 | 64.14 | 393 | 72.24 |  |
| **Among those with insurance reimbursement (n=393)** | | | | | | | | |
| **Location of insurance settlement** | 0=None-residential location | 1 | 0.51 | 107 | 54.87 | 108 | 27.48 | <0.001 |
|  | 1=Residential location | 197 | 99.49 | 88 | 45.13 | 285 | 72.52 |  |
| **Participants with the highest household income (n=2385)** | | | | | | | | |
| **Hospitalisation** | 0=No | 772 | 74.95 | 991 | 73.14 | 1,763 | 73.92 | 0.317 |
|  | 1=Yes | 258 | 25.05 | 364 | 26.86 | 622 | 26.08 |  |
| **Among those hospitalised (n=622)** | | | | | | | | |
| **Location of admitted hospital** | 0=None-residential location | 32 | 12.40 | 117 | 32.14 | 149 | 23.95 | <0.001 |
|  | 1=Residential location | 226 | 87.60 | 247 | 67.86 | 473 | 76.05 |  |
| **Insurance reimbursement** | 0=No | 32 | 14.35 | 99 | 40.57 | 131 | 28.05 | <0.001 |
|  | 1=Yes | 191 | 85.65 | 145 | 59.43 | 336 | 71.95 |  |
| **Among those with insurance reimbursement (n=336)** | | | | | | | | |
| **Location of insurance settlement** | 0=None-residential location | 1 | 0.52 | 100 | 68.97 | 101 | 30.06 | <0.001 |
|  | 1=Residential location | 190 | 99.48 | 45 | 31.03 | 235 | 69.94 |  |

# Table S3. Hospital expenses shared between insurance and out-of-pocket payments: subgroup analyses in participants divided by per capita monthly household income (in quintiles)

| **Variable** | **Measurement unit** | **with local insurance** | | **with non-local insurance** | | **Total** | | ***P*** |
| --- | --- | --- | --- | --- | --- | --- | --- | --- |
|  |  | **Median** | **Mean** | **Median** | **Mean** | **Median** | **Mean** |  |
|  |  | **(IQR)** | **(SD)** | **(IQR)** | **(SD)** | **(IQR)** | **(SD)** |  |
| **Participants with the lowest household income (n=634)** | | | | | | | | |
| **Total hospital expenditure** | Yuan | 7600 |  | 8000 |  | 7762.5 |  | 0.322 |
|  |  | (8740) |  | (8000) |  | (8300) |  |  |
| **Out-of-pocket payments** | Yuan | 3000 |  | 4750 |  | 4000 |  | <0.001 |
|  |  | (4500) |  | (5600) |  | (5500) |  |  |
| **Reimbursement ratio of hospital expenditure** | % |  | 0.53 |  | 0.40 |  | 0.44 | <0.001 |
|  |  |  | (0.21) |  | (0.22) |  | (0.22) |  |
| **Participants with the lower household income (n=439)** | | | | | | | | |
| **Total hospital expenditure** | Yuan | 8000 |  | 8000 |  | 8000 |  | 0.503 |
|  |  | (6100) |  | (7000) |  | (7000) |  |  |
| **Out-of-pocket payments** | Yuan | 3000 |  | 4200 |  | 4000 |  | <0.001 |
|  |  | (3750) |  | (4560) |  | (4500) |  |  |
| **Reimbursement ratio of hospital expenditure** | % |  | 0.54 |  | 0.42 |  | 0.47 | <0.001 |
|  |  |  | (0.20) |  | (0.23) |  | (0.22) |  |
| **Participants with the middle household income (n=407)** | | | | | | | | |
| **Total hospital expenditure** | Yuan | 7000 |  | 8000 |  | 8000 |  | 0.001 |
|  |  | (5800) |  | (6829) |  | (7000) |  |  |
| **Out-of-pocket payments** | Yuan | 3000 |  | 5000 |  | 3600 |  | <0.001 |
|  |  | (3400) |  | (5000) |  | (4700) |  |  |
| **Reimbursement ratio of hospital expenditure** | % |  | 0.58 |  | 0.40 |  | 0.48 | <0.001 |
|  |  |  | (0.21) |  | (0.22) |  | (0.23) |  |
| **Participants with the higher household income (n=381)** | | | | | | | | |
| **Total hospital expenditure** | Yuan | 7550 |  | 8600 |  | 8000 |  | 0.025 |
|  |  | (6400) |  | (10000) |  | (7800) |  |  |
| **Out-of-pocket payments** | Yuan | 3000 |  | 4600 |  | 3500 |  | <0.001 |
|  |  | (3200) |  | (6600) |  | (5000) |  |  |
| **Reimbursement ratio of hospital expenditure** | % |  | 0.55 |  | 0.44 |  | 0.50 | <0.001 |
|  |  |  | (0.21) |  | (0.24) |  | (0.23) |  |
| **Participants with the highest household income (n=325)** | | | | | | | | |
| **Total hospital expenditure** | Yuan | 8000 |  | 9140 |  | 8500 |  | 0.032 |
|  |  | (7000) |  | (8500) |  | (6700) |  |  |
| **Out-of-pocket payments** | Yuan | 3000 |  | 5000 |  | 4000 |  | <0.001 |
|  |  | (3500) |  | (7000) |  | (5000) |  |  |
| **Reimbursement ratio of hospital expenditure** | % |  | 0.57 |  | 0.41 |  | 0.50 | <0.001 |
|  |  |  | (0.22) |  | (0.24) |  | (0.24) |  |

# Table S4. Logit regression on hospitalisation: subgroup analyses in study participants divided by household income (in quintiles)

**(a)**

| **Variable** | **Hospitalisation (n=15302)** | | | | | | | | | | | | | | | | | | | |
| --- | --- | --- | --- | --- | --- | --- | --- | --- | --- | --- | --- | --- | --- | --- | --- | --- | --- | --- | --- | --- |
|  | **Lowest income (n=4300)** | | | | **Lower income (n=3241)** | | | | **Middle income (n=2855)** | | | | **Higher income (n=2548)** | | | | **Highest income (n=2385)** | | | |
|  | **OR** | **P value** | **95%CI** | | **OR** | **P value** | **95%CI** | | **OR** | **P value** | **95%CI** | | **OR** | **P value** | **95%CI** | | **OR** | **P value** | **95%CI** | |
| **Type of health insurance** (**Ref:** BMIURR) | | | | | | | | | | | | | | | | | | | | |
| BMIUE | 0.846 | 0.232 | 0.643 | 1.113 | 1.029 | 0.832 | 0.789 | 1.343 | 0.777 | 0.061 | 0.596 | 1.012 | 1.174 | 0.243 | 0.897 | 1.536 | 0.912 | 0.535 | 0.681 | 1.221 |
| Free medical care | 1.804 | 0.454 | 0.386 | 8.446 | 0.390 | 0.239 | 0.081 | 1.872 | 0.894 | 0.893 | 0.174 | 4.596 | 0.795 | 0.686 | 0.261 | 2.423 | 0.667 | 0.428 | 0.245 | 1.814 |
| **Local fund** (**Ref**: No) | | | | | | | | | | | | | | | | | | | | |
| Yes | **1.270** | **0.013** | 1.051 | 1.535 | 1.250 | 0.053 | 0.997 | 1.568 | **1.771** | **<0.001** | 1.387 | 2.261 | 1.207 | 0.163 | 0.927 | 1.572 | 1.127 | 0.417 | 0.845 | 1.503 |
| **Gender** (**Ref**: Female) | | | | | | | | | | | | | | | | | | | | |
| Male | **0.731** | **<0.001** | 0.634 | 0.845 | **0.578** | **<0.001** | 0.486 | 0.687 | **0.602** | **<0.001** | 0.501 | 0.723 | **0.625** | **<0.001** | 0.513 | 0.761 | **0.599** | **<0.001** | 0.489 | 0.734 |
| **Age** (**Ref**: 15-29 years) | | | | | | | | | | | | | | | | | | | | |
| 30-44 | **0.362** | **<0.001** | 0.291 | 0.451 | **0.496** | **<0.001** | 0.398 | 0.619 | **0.493** | **<0.001** | 0.391 | 0.621 | **0.482** | **<0.001** | 0.378 | 0.614 | **0.687** | **0.004** | 0.532 | 0.887 |
| 45-59 | **0.349** | **<0.001** | 0.273 | 0.447 | **0.385** | **<0.001** | 0.295 | 0.502 | **0.405** | **<0.001** | 0.302 | 0.544 | **0.450** | **<0.001** | 0.326 | 0.621 | 0.759 | 0.136 | 0.528 | 1.091 |
| 60 and above | **0.442** | **<0.001** | 0.330 | 0.592 | **0.643** | **0.021** | 0.442 | 0.937 | 0.758 | 0.174 | 0.508 | 1.131 | **0.629** | **0.038** | 0.406 | 0.975 | 0.878 | 0.591 | 0.547 | 1.410 |
| **Educational attainment** (**Ref:** Primary school or below) | | | | | | | | | | | | | | | | | | | | |
| Junior high | 1.053 | 0.537 | 0.894 | 1.239 | 1.107 | 0.351 | 0.894 | 1.370 | **1.364** | **0.020** | 1.051 | 1.771 | 0.966 | 0.828 | 0.704 | 1.325 | 1.455 | 0.083 | 0.952 | 2.224 |
| ≥Senior high | **1.449** | **0.001** | 1.174 | 1.788 | **1.400** | **0.007** | 1.096 | 1.788 | **1.505** | **0.005** | 1.129 | 2.005 | 0.991 | 0.958 | 0.715 | 1.374 | **1.566** | **0.041** | 1.018 | 2.408 |
| **Marital status** (**Ref:** Single) | | | | | | | | | | | | | | | | | | | | |
| Married/ Cohabiting | **4.926** | **<0.001** | 2.859 | 8.485 | **3.387** | **<0.001** | 1.984 | 5.780 | **6.181** | **<0.001** | 3.901 | 9.796 | **5.862** | **<0.001** | 4.088 | 8.405 | **5.897** | **<0.001** | 4.347 | 8.001 |
| Divorced/ Widowed | **4.371** | **<0.001** | 2.338 | 8.169 | **3.030** | **0.002** | 1.504 | 6.102 | **2.445** | **0.015** | 1.186 | 5.042 | **3.453** | **<0.001** | 1.814 | 6.573 | **3.745** | **<0.001** | 2.113 | 6.639 |
| **Self-rating of health** (**Ref**: Poor) | | | | | | | | | | | | | | | | | | | | |
| General | **0.488** | **<0.001** | 0.406 | 0.587 | **0.648** | **0.002** | 0.495 | 0.849 | **0.452** | **<0.001** | 0.330 | 0.617 | **0.296** | **<0.001** | 0.209 | 0.420 | **0.486** | **0.002** | 0.309 | 0.764 |
| Good | **0.610** | **<0.001** | 0.502 | 0.740 | 0.890 | 0.392 | 0.681 | 1.163 | **0.533** | **<0.001** | 0.391 | 0.726 | **0.297** | **<0.001** | 0.210 | 0.421 | **0.484** | **0.002** | 0.307 | 0.764 |
| **Distance of migration** (**Ref**: Inter-county) | | | | | | | | | | | | | | | | | | | | |
| Inter-city | **0.810** | **0.019** | 0.679 | 0.967 | 0.800 | 0.051 | 0.639 | 1.001 | 1.072 | 0.583 | 0.837 | 1.373 | 1.012 | 0.929 | 0.784 | 1.306 | 0.876 | 0.377 | 0.652 | 1.176 |
| Inter-province | **0.695** | **<0.001** | 0.575 | 0.840 | **0.709** | **0.004** | 0.561 | 0.897 | 0.810 | 0.103 | 0.630 | 1.043 | **0.590** | **<0.001** | 0.449 | 0.776 | **0.638** | **0.003** | 0.472 | 0.861 |
| **Years of residing in migration destination** (**Ref**: <1) | | | | | | | | | | | | | | | | | | | | |
| 1-4 | 0.915 | 0.464 | 0.721 | 1.161 | 1.015 | 0.913 | 0.779 | 1.323 | 0.863 | 0.301 | 0.654 | 1.141 | 1.045 | 0.756 | 0.790 | 1.384 | 0.910 | 0.535 | 0.675 | 1.226 |
| 5-9 | 0.933 | 0.576 | 0.732 | 1.189 | 0.890 | 0.424 | 0.669 | 1.184 | 0.885 | 0.422 | 0.658 | 1.191 | 0.886 | 0.455 | 0.645 | 1.217 | 0.789 | 0.172 | 0.561 | 1.109 |
| 10 and above | 0.854 | 0.201 | 0.670 | 1.088 | 0.929 | 0.630 | 0.690 | 1.252 | 0.886 | 0.449 | 0.648 | 1.212 | 0.998 | 0.991 | 0.711 | 1.401 | 1.068 | 0.727 | 0.739 | 1.544 |
| **Migration destination** (**Ref**: Pearl River Delta) | | | | | | | | | | | | | | | | | | | | |
| Yangtze River delta | 1.090 | 0.629 | 0.769 | 1.544 | 1.273 | 0.196 | 0.883 | 1.838 | **1.483** | **0.039** | 1.021 | 2.153 | 0.964 | 0.870 | 0.619 | 1.499 | 0.966 | 0.884 | 0.606 | 1.539 |
| Circum-Bohai Sea | 1.050 | 0.789 | 0.735 | 1.500 | **1.479** | **0.046** | 1.008 | 2.171 | **1.513** | **0.045** | 1.010 | 2.266 | 1.007 | 0.977 | 0.638 | 1.589 | 0.868 | 0.562 | 0.538 | 1.400 |
| others | 1.163 | 0.355 | 0.845 | 1.601 | **1.626** | **0.004** | 1.172 | 2.255 | **1.761** | **0.001** | 1.251 | 2.479 | 1.308 | 0.188 | 0.877 | 1.953 | 1.303 | 0.218 | 0.855 | 1.987 |
| **R^2^** | 0.051 | | | | 0.061 | | | | 0.077 | | | | 0.102 | | | | 0.104 | | | |

**(b)**

| **Variable** | **Local (residential) admission (n=4423)** | | | | | | | | | | | | | | | | | | | |
| --- | --- | --- | --- | --- | --- | --- | --- | --- | --- | --- | --- | --- | --- | --- | --- | --- | --- | --- | --- | --- |
|  | **Lowest income (n=1323)** | | | | **Lower income (n=940)** | | | | **Middle income (n=803)** | | | | **Higher income (n=735)** | | | | **Highest income (n=622)** | | | |
|  | **OR** | **P value** | **95%CI** | | **OR** | **P value** | **95%CI** | | **OR** | **P value** | **95%CI** | | **OR** | **P value** | **95%CI** | | **OR** | **P value** | **95%CI** | |
| **Type of health insurance** (**Ref:** BMIURR) | | | | | | | | | | | | | | | | | | | | |
| BMIUE | 0.660 | 0.156 | 0.371 | 1.172 | 0.612 | 0.065 | 0.363 | 1.030 | 0.944 | 0.824 | 0.565 | 1.575 | 1.309 | 0.330 | 0.761 | 2.249 | 0.730 | 0.263 | 0.420 | 1.267 |
| Free medical care | 0.078 | 0.055 | 0.006 | 1.059 | 0.063 | 0.067 | 0.003 | 1.219 | 0.194 | 0.257 | 0.011 | 3.312 | 1.593 | 0.705 | 0.143 | 17.720 | 0.487 | 0.440 | 0.079 | 3.023 |
| **Local fund** (**Ref**: No) | | | | | | | | | | | | | | | | | | | | |
| Yes | **4.759** | **<0.001** | 3.087 | 7.337 | **4.025** | **<0.001** | 2.497 | 6.488 | **1.827** | **0.011** | 1.151 | 2.901 | **2.305** | **0.002** | 1.366 | 3.890 | **4.114** | **<0.001** | 2.308 | 7.334 |
| **Gender** (**Ref**: Female) | | | | | | | | | | | | | | | | | | | | |
| Male | 0.952 | 0.736 | 0.714 | 1.268 | 0.876 | 0.469 | 0.613 | 1.253 | 1.177 | 0.418 | 0.793 | 1.747 | 1.050 | 0.821 | 0.691 | 1.595 | 0.826 | 0.394 | 0.533 | 1.282 |
| **Age** (**Ref**: 15-29 years) | | | | | | | | | | | | | | | | | | | | |
| 30-44 | 0.677 | 0.064 | 0.448 | 1.023 | 0.979 | 0.921 | 0.644 | 1.488 | 0.807 | 0.355 | 0.512 | 1.271 | 0.789 | 0.346 | 0.483 | 1.291 | 0.907 | 0.709 | 0.545 | 1.511 |
| 45-59 | 0.747 | 0.245 | 0.457 | 1.222 | 1.089 | 0.749 | 0.645 | 1.841 | 0.819 | 0.500 | 0.458 | 1.464 | 0.580 | 0.092 | 0.308 | 1.093 | 0.836 | 0.618 | 0.413 | 1.692 |
| 60 and above | 0.849 | 0.575 | 0.478 | 1.507 | 1.205 | 0.603 | 0.596 | 2.436 | 0.922 | 0.844 | 0.412 | 2.064 | 0.895 | 0.798 | 0.383 | 2.091 | 1.407 | 0.457 | 0.572 | 3.461 |
| **Educational attainment** (**Ref:** Primary school or below) | | | | | | | | | | | | | | | | | | | | |
| Junior high | 1.271 | 0.128 | 0.934 | 1.730 | 1.151 | 0.499 | 0.765 | 1.731 | 0.971 | 0.907 | 0.590 | 1.597 | 1.005 | 0.986 | 0.567 | 1.781 | 0.940 | 0.884 | 0.409 | 2.161 |
| ≥Senior high | 1.480 | 0.056 | 0.990 | 2.212 | 1.376 | 0.182 | 0.861 | 2.198 | 1.621 | 0.095 | 0.919 | 2.859 | 1.428 | 0.250 | 0.779 | 2.620 | 0.849 | 0.696 | 0.372 | 1.936 |
| **Marital status** (**Ref:** Single) | | | | | | | | | | | | | | | | | | | | |
| Married/ Cohabiting | 2.890 | 0.051 | 0.995 | 8.389 | 0.444 | 0.241 | 0.114 | 1.726 | 1.484 | 0.411 | 0.579 | 3.807 | 1.193 | 0.650 | 0.557 | 2.558 | 1.310 | 0.425 | 0.674 | 2.544 |
| Divorced/ Widowed | 2.130 | 0.219 | 0.638 | 7.110 | 0.315 | 0.150 | 0.065 | 1.519 | 4.305 | 0.095 | 0.775 | 23.919 | 1.233 | 0.751 | 0.338 | 4.502 | 2.171 | 0.231 | 0.611 | 7.716 |
| **Self-rating of health** (**Ref**: Poor) | | | | | | | | | | | | | | | | | | | | |
| General | 1.150 | 0.425 | 0.816 | 1.621 | 1.858 | 0.013 | 1.139 | 3.033 | **2.881** | **<0.001** | 1.643 | 5.052 | 1.035 | 0.907 | 0.582 | 1.840 | 1.083 | 0.837 | 0.508 | 2.307 |
| Good | 1.343 | 0.125 | 0.921 | 1.959 | **2.562** | **<0.001** | 1.558 | 4.211 | **3.192** | **<0.001** | 1.818 | 5.604 | 1.211 | 0.528 | 0.668 | 2.193 | 1.710 | 0.178 | 0.784 | 3.730 |
| **Distance of migration** (**Ref**: Inter-county) | | | | | | | | | | | | | | | | | | | | |
| Inter-city | 0.804 | 0.199 | 0.577 | 1.121 | **0.571** | **0.012** | 0.369 | 0.884 | **0.479** | **0.005** | 0.286 | 0.803 | 0.626 | 0.074 | 0.374 | 1.046 | **1.848** | **0.028** | 1.070 | 3.192 |
| Inter-province | **0.677** | **0.035** | 0.471 | 0.972 | **0.529** | **0.007** | 0.335 | 0.837 | **0.368** | **<0.001** | 0.216 | 0.626 | **0.531** | **0.025** | 0.306 | 0.923 | 1.154 | 0.610 | 0.665 | 2.002 |
| **Years of residing in migration destination** (**Ref**: <1) | | | | | | | | | | | | | | | | | | | | |
| 1-4 | **2.893** | **<0.001** | 1.923 | 4.352 | **2.440** | **<0.001** | 1.540 | 3.867 | **2.536** | **<0.001** | 1.551 | 4.149 | **2.271** | **0.002** | 1.362 | 3.788 | 1.652 | 0.079 | 0.943 | 2.891 |
| 5-9 | **3.805** | **<0.001** | 2.479 | 5.839 | **2.241** | **0.002** | 1.343 | 3.740 | **3.746** | **<0.001** | 2.150 | 6.524 | **1.852** | **0.037** | 1.039 | 3.301 | 1.471 | 0.247 | 0.765 | 2.827 |
| 10 and above | **2.379** | **<0.001** | 1.547 | 3.656 | **2.589** | **0.001** | 1.483 | 4.519 | **3.120** | **<0.001** | 1.740 | 5.596 | **3.455** | **<0.001** | 1.779 | 6.713 | 1.885 | 0.078 | 0.931 | 3.815 |
| **Migration destination** (**Ref**: Pearl River Delta) | | | | | | | | | | | | | | | | | | | | |
| Yangtze River delta | 0.820 | 0.556 | 0.424 | 1.586 | 0.950 | 0.890 | 0.461 | 1.957 | 0.598 | 0.183 | 0.281 | 1.274 | 1.174 | 0.734 | 0.465 | 2.964 | 1.412 | 0.466 | 0.559 | 3.564 |
| Circum-Bohai Sea | 1.156 | 0.682 | 0.578 | 2.310 | 1.633 | 0.227 | 0.737 | 3.619 | **3.264** | **0.015** | 1.256 | 8.479 | 1.805 | 0.246 | 0.665 | 4.901 | 1.122 | 0.806 | 0.449 | 2.802 |
| others | 0.976 | 0.938 | 0.524 | 1.818 | 0.946 | 0.868 | 0.493 | 1.815 | 0.827 | 0.598 | 0.409 | 1.673 | 1.092 | 0.840 | 0.467 | 2.553 | 1.690 | 0.209 | 0.746 | 3.829 |
| **R^2^** | 0.088 | | | | 0.090 | | | | 0.109 | | | | 0.089 | | | | 0.087 | | | |

**(c)**

| **Variable** | **Insurance reimbursement (n=3175)** | | | | | | | | | | | | | | | | | | | |
| --- | --- | --- | --- | --- | --- | --- | --- | --- | --- | --- | --- | --- | --- | --- | --- | --- | --- | --- | --- | --- |
|  | **Lowest income (n=938)** | | | | **Lower income (n=661)** | | | | **Middle income (n=573)** | | | | **Higher income (n=540)** | | | | **Highest income (n=463)** | | | |
|  | **OR** | **P value** | **95%CI** | | **OR** | **P value** | **95%CI** | | **OR** | **P value** | **95%CI** | | **OR** | **P value** | **95%CI** | | **OR** | **P value** | **95%CI** | |
| **Type of health insurance** (**Ref:** BMIURR) | | | | | | | | | | | | | | | | | | | | |
| BMIUE | 1.349 | 0.338 | 0.731 | 2.489 | **2.144** | **0.015** | 1.159 | 3.966 | **2.359** | **0.009** | 1.236 | 4.502 | 1.329 | 0.391 | 0.694 | 2.544 | 1.292 | 0.443 | 0.671 | 2.489 |
| Free medical care | - | - | - | - | - | - | - | - | - | - | - | - | - | - | - | - | - | - | - | - |
| **Local fund** (**Ref**: No) | | | | | | | | | | | | | | | | | | | | |
| Yes | **2.277** | **<0.001** | 1.505 | 3.446 | **2.391** | **0.001** | 1.463 | 3.907 | **2.954** | **<0.001** | 1.668 | 5.230 | **3.488** | **<0.001** | 1.873 | 6.494 | **3.919** | **<0.001** | 2.043 | 7.518 |
| **Gender** (**Ref**: Female) | | | | | | | | | | | | | | | | | | | | |
| Male | 0.818 | 0.261 | 0.576 | 1.161 | 0.871 | 0.528 | 0.566 | 1.339 | 0.937 | 0.790 | 0.580 | 1.514 | **1.985** | **0.010** | 1.180 | 3.339 | 1.048 | 0.867 | 0.608 | 1.805 |
| **Age** (**Ref**: 15-29 years) | | | | | | | | | | | | | | | | | | | | |
| 30-44 | 1.046 | 0.840 | 0.678 | 1.613 | 1.060 | 0.802 | 0.673 | 1.669 | 1.049 | 0.853 | 0.632 | 1.741 | 1.353 | 0.263 | 0.797 | 2.298 | 1.567 | 0.117 | 0.894 | 2.746 |
| 45-59 | **1.745** | **0.047** | 1.006 | 3.025 | 1.680 | 0.093 | 0.917 | 3.078 | **2.080** | **0.048** | 1.007 | 4.299 | 1.571 | 0.275 | 0.698 | 3.536 | **2.797** | **0.027** | 1.125 | 6.953 |
| 60 and above | 1.956 | 0.053 | 0.992 | 3.858 | **4.435** | **0.006** | 1.545 | 12.730 | 1.638 | 0.339 | 0.596 | 4.505 | **6.338** | **0.004** | 1.806 | 22.243 | 2.457 | 0.133 | 0.761 | 7.930 |
| **Educational attainment** (**Ref:** Primary school or below) | | | | | | | | | | | | | | | | | | | | |
| Junior high | 1.289 | 0.187 | 0.884 | 1.879 | 0.998 | 0.993 | 0.606 | 1.642 | 1.256 | 0.492 | 0.656 | 2.403 | 1.140 | 0.744 | 0.519 | 2.502 | 1.447 | 0.482 | 0.517 | 4.050 |
| ≥Senior high | 1.335 | 0.216 | 0.844 | 2.113 | 1.289 | 0.364 | 0.745 | 2.230 | 1.421 | 0.321 | 0.710 | 2.846 | 1.609 | 0.248 | 0.718 | 3.607 | 1.858 | 0.239 | 0.663 | 5.209 |
| **Marital status** (**Ref:** Single) | | | | | | | | | | | | | | | | | | | | |
| Married/ Cohabiting | 1.201 | 0.799 | 0.293 | 4.923 | 0.990 | 0.987 | 0.281 | 3.489 | 0.313 | 0.098 | 0.079 | 1.240 | **2.600** | **0.038** | 1.054 | 6.413 | 1.034 | 0.931 | 0.481 | 2.224 |
| Divorced/ Widowed | 1.264 | 0.777 | 0.250 | 6.394 | 0.674 | 0.647 | 0.125 | 3.634 | 0.405 | 0.378 | 0.055 | 3.011 | **11.538** | **0.041** | 1.103 | 120.704 | 1.638 | 0.556 | 0.316 | 8.476 |
| **Self-rating of health** (**Ref**: Poor) | | | | | | | | | | | | | | | | | | | | |
| General | 0.811 | 0.369 | 0.514 | 1.280 | 1.137 | 0.716 | 0.569 | 2.274 | 0.757 | 0.513 | 0.329 | 1.743 | 0.859 | 0.707 | 0.390 | 1.896 | 0.456 | 0.227 | 0.128 | 1.629 |
| Good | **0.473** | **0.002** | 0.297 | 0.754 | 0.883 | 0.720 | 0.446 | 1.746 | 0.596 | 0.222 | 0.260 | 1.368 | 0.841 | 0.668 | 0.382 | 1.853 | 0.371 | 0.128 | 0.104 | 1.330 |
| **Distance of migration** (**Ref**: Inter-county) | | | | | | | | | | | | | | | | | | | | |
| Inter-city | **0.570** | **0.007** | 0.380 | 0.856 | 0.763 | 0.276 | 0.470 | 1.241 | 0.575 | 0.059 | 0.323 | 1.021 | **0.553** | **0.045** | 0.310 | 0.987 | 1.520 | 0.206 | 0.794 | 2.908 |
| Inter-province | **0.292** | **<0.001** | 0.189 | 0.453 | **0.508** | **0.008** | 0.308 | 0.839 | 0.567 | 0.062 | 0.313 | 1.029 | **0.325** | **0.001** | 0.172 | 0.612 | 0.734 | 0.364 | 0.377 | 1.431 |
| **Years of residing in migration destination** (**Ref**: <1) | | | | | | | | | | | | | | | | | | | | |
| 1-4 | 1.108 | 0.718 | 0.633 | 1.940 | 0.866 | 0.644 | 0.469 | 1.596 | 0.752 | 0.419 | 0.376 | 1.502 | 0.665 | 0.250 | 0.331 | 1.334 | 1.567 | 0.176 | 0.817 | 3.003 |
| 5-9 | 1.378 | 0.273 | 0.777 | 2.441 | 0.984 | 0.961 | 0.503 | 1.923 | 0.567 | 0.123 | 0.276 | 1.167 | 1.076 | 0.860 | 0.477 | 2.425 | **2.410** | **0.030** | 1.090 | 5.326 |
| 10 and above | 1.080 | 0.803 | 0.591 | 1.974 | 0.985 | 0.968 | 0.481 | 2.019 | 0.897 | 0.793 | 0.398 | 2.020 | 0.533 | 0.149 | 0.226 | 1.253 | 1.764 | 0.197 | 0.744 | 4.181 |
| **Migration destination** (**Ref**: Pearl River Delta) | | | | | | | | | | | | | | | | | | | | |
| Yangtze River delta | 0.558 | 0.126 | 0.264 | 1.178 | 0.520 | 0.135 | 0.221 | 1.225 | 0.769 | 0.578 | 0.304 | 1.942 | 0.495 | 0.202 | 0.168 | 1.457 | 0.963 | 0.950 | 0.295 | 3.146 |
| Circum-Bohai Sea | **0.427** | **0.030** | 0.198 | 0.920 | 0.521 | 0.141 | 0.219 | 1.241 | 0.547 | 0.198 | 0.218 | 1.370 | **0.283** | **0.022** | 0.096 | 0.835 | 0.914 | 0.884 | 0.274 | 3.054 |
| others | 0.842 | 0.627 | 0.420 | 1.686 | 0.877 | 0.740 | 0.405 | 1.899 | 1.329 | 0.497 | 0.585 | 3.023 | 0.760 | 0.589 | 0.281 | 2.057 | 0.762 | 0.610 | 0.268 | 2.166 |
| **R^2^** | 0.115 | | | | 0.112 | | | | 0.118 | | | | 0.154 | | | | 0.148 | | | |

**(d)**

| **Variable** | **Local (residential) settlement of insurance claims (n=2226)** | | | | | | | | | | | | | | | | | | | |
| --- | --- | --- | --- | --- | --- | --- | --- | --- | --- | --- | --- | --- | --- | --- | --- | --- | --- | --- | --- | --- |
|  | **Lowest income (n=642)** | | | | **Lower income (n=439)** | | | | **Middle income (n=416)** | | | | **Higher income (n=393)** | | | | **Highest income (n=336)** | | | |
|  | **OR** | **P value** | **95%CI** | | **OR** | **P value** | **95%CI** | | **OR** | **P value** | **95%CI** | | **OR** | **P value** | **95%CI** | | **OR** | **P value** | **95%CI** | |
| **Type of health insurance** (**Ref:** BMIURR) | | | | | | | | | | | | | | | | | | | | |
| BMIUE | 0.869 | 0.808 | 0.279 | 2.707 | **4.956** | **0.004** | 1.679 | 14.631 | 2.311 | 0.053 | 0.991 | 5.393 | 0.437 | 0.148 | 0.142 | 1.343 | 2.375 | 0.141 | 0.750 | 7.518 |
| Free medical care | - | - | - | - | - | - | - | - | - | - | - | - | 0.192 | 0.264 | 0.011 | 3.477 | 0.032 | 0.412 | <0.001 | 120.046 |
| **Local fund** (**Ref**: No) | | | | | | | | | | | | | | | | | | | | |
| Yes | **146.176** | **<0.001** | 46.696 | 457.588 | **117.9** | **<0.001** | 32.345 | 429.746 | **104.199** | **<0.001** | 35.639 | 304.651 | **1606.301** | **<0.001** | 129.707 | 19893 | **1500.754** | **<0.001** | 141.214 | 15949 |
| **Gender** (**Ref**: Female) | | | | | | | | | | | | | | | | | | | | |
| Male | 0.910 | 0.705 | 0.559 | 1.482 | 1.08 | 0.821 | 0.556 | 2.100 | 1.037 | 0.919 | 0.513 | 2.099 | 1.06 | 0.879 | 0.503 | 2.232 | 1.759 | 0.282 | 0.629 | 4.920 |
| **Age** (**Ref**: 15-29 years) | | | | | | | | | | |  |  |  |  |  |  |  |  |  |  |
| 30-44 | 1.311 | 0.436 | 0.664 | 2.587 | 1.086 | 0.833 | 0.504 | 2.339 | 1.905 | 0.121 | 0.843 | 4.304 | 2.008 | 0.154 | 0.77 | 5.238 | **5.657** | **0.011** | 1.491 | 21.465 |
| 45-59 | 1.563 | 0.276 | 0.700 | 3.490 | 1.150 | 0.773 | 0.445 | 2.971 | 2.519 | 0.085 | 0.882 | 7.194 | 1.233 | 0.738 | 0.362 | 4.206 | 5.305 | 0.073 | 0.856 | 32.881 |
| 60 and above | 2.504 | 0.055 | 0.982 | 6.383 | 1.003 | 0.997 | 0.289 | 3.473 | **5.203** | **0.021** | 1.276 | 21.217 | **5.448** | **0.040** | 1.083 | 27.398 | **8.221** | **0.050** | 1.004 | 67.285 |
| **Educational attainment** (**Ref:** Primary school or below) | | | | | | | | | |  |  |  |  |  |  |  |  |  |  |  |
| Junior high | 0.852 | 0.553 | 0.502 | 1.445 | 1.539 | 0.260 | 0.727 | 3.257 | 0.545 | 0.208 | 0.212 | 1.401 | 0.846 | 0.762 | 0.288 | 2.490 | 1.340 | 0.735 | 0.246 | 7.312 |
| ≥Senior high | 1.155 | 0.673 | 0.591 | 2.259 | 0.842 | 0.684 | 0.369 | 1.922 | 0.919 | 0.870 | 0.334 | 2.526 | 1.084 | 0.884 | 0.368 | 3.187 | 1.307 | 0.757 | 0.239 | 7.144 |
| **Marital status** (**Ref:** Single) | | | | | | | | | | | | | | | | | | | | |
| Married/ Cohabiting | <0.001 | 0.978 | <0.001 | >0.99 | 1.965 | 0.488 | 0.291 | 13.276 | 0.541 | 0.510 | 0.087 | 3.369 | 0.420 | 0.396 | 0.057 | 3.107 | 0.208 | 0.082 | 0.035 | 1.224 |
| Divorced/Widowed | <0.001 | 0.977 | <0.001 | >0.99 | 1.143 | 0.920 | 0.084 | 15.612 | 0.160 | 0.186 | 0.011 | 2.420 | 0.811 | 0.872 | 0.063 | 10.501 | 0.060 | 0.077 | 0.003 | 1.364 |
| **Self-rating of health** (**Ref**: Poor) | | | | | | | | | | | | | | | | | | | | |
| General | 1.145 | 0.644 | 0.645 | 2.032 | 0.783 | 0.609 | 0.306 | 2.000 | 1.563 | 0.388 | 0.567 | 4.312 | 0.647 | 0.402 | 0.233 | 1.792 | 1.05 | 0.947 | 0.256 | 4.311 |
| Good | 1.205 | 0.563 | 0.641 | 2.266 | 0.634 | 0.368 | 0.235 | 1.709 | 1.721 | 0.329 | 0.578 | 5.123 | 0.664 | 0.459 | 0.225 | 1.959 | 1.662 | 0.524 | 0.348 | 7.936 |
| **Distance of migration** (**Ref**: Inter-county) | | | | | | | | | | | | | | | | | | | | |
| Inter-city | **0.210** | **<0.001** | 0.130 | 0.340 | **0.430** | **0.011** | 0.224 | 0.825 | **0.222** | **<0.001** | 0.106 | 0.469 | **0.256** | **<0.001** | 0.123 | 0.531 | **0.257** | **0.016** | 0.086 | 0.774 |
| Inter-province | **0.218** | **<0.001** | 0.114 | 0.42 | **0.141** | **<0.001** | 0.061 | 0.325 | **0.154** | **<0.001** | 0.064 | 0.373 | **0.081** | **<0.001** | 0.027 | 0.243 | **0.050** | **<0.001** | 0.011 | 0.237 |
| **Years of residing in migration destination** (**Ref**: <1) | | | | | | | | | | | | | | | | | | | | |
| 1-4 | 0.674 | 0.344 | 0.298 | 1.526 | 0.779 | 0.605 | 0.302 | 2.010 | 1.486 | 0.452 | 0.530 | 4.168 | 1.248 | 0.662 | 0.462 | 3.373 | 1.408 | 0.638 | 0.339 | 5.851 |
| 5-9 | 0.623 | 0.260 | 0.274 | 1.419 | 0.960 | 0.938 | 0.344 | 2.678 | 1.923 | 0.230 | 0.662 | 5.590 | 1.200 | 0.752 | 0.388 | 3.714 | 0.954 | 0.954 | 0.193 | 4.707 |
| 10 and above | 0.599 | 0.245 | 0.253 | 1.421 | 0.858 | 0.789 | 0.28 | 2.634 | 1.128 | 0.838 | 0.356 | 3.579 | 0.833 | 0.764 | 0.253 | 2.744 | 0.735 | 0.707 | 0.147 | 3.675 |
| **Migration destination** (**Ref**: Pearl River Delta) | | | | | | | | | | | | | | | | | | | | |
| Yangtze River delta | 0.840 | 0.817 | 0.192 | 3.674 | 2.334 | 0.360 | 0.381 | 14.305 | 0.295 | 0.206 | 0.045 | 1.959 | 2.683 | 0.513 | 0.139 | 51.709 | 0.351 | 0.497 | 0.017 | 7.189 |
| Circum-Bohai Sea | 1.429 | 0.613 | 0.358 | 5.698 | 2.372 | 0.317 | 0.437 | 12.864 | 0.778 | 0.790 | 0.123 | 4.905 | 5.031 | 0.261 | 0.301 | 84.064 | 0.464 | 0.600 | 0.026 | 8.192 |
| others | 2.507 | 0.152 | 0.713 | 8.812 | 3.142 | 0.140 | 0.687 | 14.376 | 1.225 | 0.808 | 0.239 | 6.273 | 3.888 | 0.325 | 0.261 | 57.971 | 0.402 | 0.506 | 0.027 | 5.899 |
| **R^2^** | 0.379 | | | | 0.433 | | | | 0.48 | | | | 0.494 | | | | 0.647 | | | |

# Table S5. Health insurance use for hospital care in BMIURR enrollees: results of logit regression models

| **Variable** | **Hospitalisation**  **(n=11555)** | | | | **Local (residential) admission (n=3314)** | | | | | **Insurance reimbursement (n=2290)** | | | | **Local (residential) settlement of insurance claims**  **(n=1474)** | | | |
| --- | --- | --- | --- | --- | --- | --- | --- | --- | --- | --- | --- | --- | --- | --- | --- | --- | --- |
|  | **OR** | **P value** | **95%CI** | | **OR** | | **P value** | **95%CI** | | **OR** | **P value** | **95%CI** | | **OR** | **P value** | **95%CI** | |
| **Local fund** (**Ref**: No) | | |  |  |  | |  |  |  |  |  |  |  |  |  |  |  |
| Yes | **1.512** | **<0.001** | 1.340 | 1.705 | **3.183** | | **<0.001** | 2.452 | 4.133 | **3.295** | **<0.001** | 2.513 | 4.320 | **95.083** | **<0.001** | 51.572 | 175.304 |
| **Gender** (**Ref**: Female) | | |  |  |  | |  |  |  |  |  |  |  |  |  |  |  |
| Male | **0.687** | **<0.001** | 0.628 | 0.752 | 0.998 | | 0.980 | 0.835 | 1.192 | 0.926 | 0.489 | 0.745 | 1.151 | 1.061 | 0.704 | 0.781 | 1.442 |
| **Age** (**Ref**: 15-29 years) | |  |  |  |  | |  |  |  |  |  |  |  |  |  |  |  |
| 30-44 | **0.450** | **<0.001** | 0.399 | 0.508 | **0.748** | | **0.010** | 0.600 | 0.933 | 1.076 | 0.554 | 0.844 | 1.373 | **1.623** | **0.013** | 1.109 | 2.376 |
| 45-59 | **0.388** | **<0.001** | 0.337 | 0.448 | 0.778 | | 0.072 | 0.592 | 1.023 | **1.756** | **0.001** | 1.271 | 2.428 | **1.986** | **0.005** | 1.234 | 3.194 |
| 60 and above | **0.539** | **<0.001** | 0.446 | 0.652 | 0.830 | | 0.303 | 0.583 | 1.183 | **1.937** | **0.004** | 1.238 | 3.030 | **3.596** | **<0.001** | 1.960 | 6.596 |
| **Educational attainment** (**Ref:** Primary school or below) | | | | | | |  |  |  |  |  |  |  |  |  |  |  |
| Junior high | **1.138** | **0.023** | 1.018 | 1.271 | 1.193 | | 0.091 | 0.972 | 1.466 | 1.130 | 0.353 | 0.873 | 1.464 | 1.062 | 0.743 | 0.740 | 1.524 |
| ≥Senior high | **1.346** | **<0.001** | 1.180 | 1.534 | **1.535** | | **0.001** | 1.202 | 1.959 | **1.356** | **0.041** | 1.013 | 1.814 | 1.206 | 0.377 | 0.796 | 1.829 |
| **Marital status** (**Ref:** Single) | | | |  |  | |  |  |  |  |  |  |  |  |  |  |  |
| Married/ Cohabiting | **5.478** | **<0.001** | 4.422 | 6.786 | 1.299 | | 0.228 | 0.849 | 1.989 | 1.041 | 0.878 | 0.624 | 1.736 | 0.576 | 0.197 | 0.249 | 1.333 |
| Divorced/  Widowed | **3.832** | **<0.001** | 2.834 | 5.181 | 1.161 | | 0.619 | 0.645 | 2.089 | 1.069 | 0.862 | 0.503 | 2.272 | **0.288** | **0.030** | 0.094 | 0.889 |
| **Monthly per capita household income ranking** (**Ref:** Lowest) | | | | | |  |  |  |  |  |  |  |  |  |  |  |  |
| Lower | 0.892 | 0.050 | 0.795 | 1.000 | 0.862 | | 0.169 | 0.698 | 1.065 | 0.927 | 0.555 | 0.721 | 1.192 | 1.066 | 0.733 | 0.738 | 1.538 |
| Middle | **0.879** | **0.043** | 0.775 | 0.996 | 0.857 | | 0.190 | 0.679 | 1.080 | 1.171 | 0.267 | 0.886 | 1.546 | 0.716 | 0.104 | 0.479 | 1.071 |
| Higher | 0.874 | 0.058 | 0.760 | 1.004 | 0.867 | | 0.271 | 0.672 | 1.118 | 0.941 | 0.691 | 0.697 | 1.271 | 1.305 | 0.225 | 0.849 | 2.005 |
| Highest | 0.971 | 0.721 | 0.828 | 1.140 | 0.964 | | 0.806 | 0.718 | 1.294 | 0.943 | 0.725 | 0.679 | 1.310 | 0.639 | 0.091 | 0.380 | 1.075 |
| **Self-rating of health** (**Ref**: Poor) | | |  |  |  | |  |  |  |  |  |  |  |  |  |  |  |
| General | **0.510** | **<0.001** | 0.447 | 0.582 | **1.376** | | **0.008** | 1.087 | 1.741 | 0.817 | 0.214 | 0.594 | 1.124 | 1.315 | 0.187 | 0.876 | 1.976 |
| Good | **0.599** | **<0.001** | 0.524 | 0.684 | **1.603** | | **<0.001** | 1.257 | 2.043 | **0.600** | **0.002** | 0.437 | 0.825 | 1.166 | 0.486 | 0.758 | 1.793 |
| **Distance of migration** (**Ref**: Inter-county) | | | |  |  | |  |  |  |  |  |  |  |  |  |  |  |
| Inter-city | **0.866** | **0.014** | 0.771 | 0.972 | **0.738** | | **0.006** | 0.595 | 0.916 | **0.688** | **0.003** | 0.535 | 0.884 | **0.282** | **<0.001** | 0.209 | 0.381 |
| Inter-province | **0.746** | **<0.001** | 0.661 | 0.841 | **0.574** | | **<0.001** | 0.458 | 0.720 | **0.354** | **<0.001** | 0.272 | 0.462 | **0.164** | **<0.001** | 0.109 | 0.247 |
| **Years of residing in migration destination** (**Ref**: <1) | | | | | | |  |  |  |  |  |  |  |  |  |  |  |
| 1-4 | 0.946 | 0.425 | 0.827 | 1.084 | **2.323** | | **<0.001** | 1.843 | 2.928 | 0.934 | 0.668 | 0.683 | 1.276 | 1.129 | 0.608 | 0.711 | 1.792 |
| 5-9 | 0.917 | 0.244 | 0.792 | 1.061 | **2.815** | | **<0.001** | 2.173 | 3.646 | 1.066 | 0.710 | 0.761 | 1.494 | 1.130 | 0.624 | 0.693 | 1.843 |
| 10 and above | 0.907 | 0.208 | 0.780 | 1.055 | **2.470** | | **<0.001** | 1.886 | 3.234 | 0.927 | 0.682 | 0.647 | 1.330 | 0.904 | 0.704 | 0.535 | 1.525 |
| **Migration destination** (**Ref**: Pearl River Delta) | | | | | | |  |  |  |  |  |  |  |  |  |  |  |
| Yangtze River delta | 1.080 | 0.468 | 0.877 | 1.329 | 0.955 | | 0.813 | 0.650 | 1.403 | **0.573** | **0.018** | 0.361 | 0.909 | 0.713 | 0.487 | 0.275 | 1.849 |
| Circum-Bohai Sea | 1.162 | 0.180 | 0.933 | 1.446 | 1.353 | | 0.155 | 0.892 | 2.053 | **0.608** | **0.039** | 0.379 | 0.974 | 1.797 | 0.174 | 0.771 | 4.186 |
| others | **1.300** | **0.006** | 1.079 | 1.567 | 1.032 | | 0.862 | 0.726 | 1.467 | 0.933 | 0.742 | 0.619 | 1.407 | **2.150** | **0.048** | 1.008 | 4.587 |
| **R^2^** | 0.0623 | | | | 0.0619 | | | | | 0.1002 | | | | 0.3449 | | | |

# Table S6. Health insurance use for hospital care in BMIUE enrollees: results of logit regression models

| **Variable** | **Hospitalisation**  **(n=3677)** | | | | **Local (residential) admission (n=1091)** | | | | | **Insurance reimbursement (n=885)** | | | | **Local (residential) settlement of insurance claims**  **(n=744)** | | | |
| --- | --- | --- | --- | --- | --- | --- | --- | --- | --- | --- | --- | --- | --- | --- | --- | --- | --- |
|  | **OR** | **P value** | **95%CI** | | **OR** | | **P value** | **95%CI** | | **OR** | **P value** | **95%CI** | | **OR** | **P value** | **95%CI** | |
| **Local fund** (**Ref**: No) | | |  |  |  | |  |  |  |  |  |  |  |  |  |  |  |
| Yes | 0.805 | 0.088 | 0.628 | 1.033 | **4.221** | | **<0.001** | 2.709 | 6.576 | 1.556 | 0.176 | 0.820 | 2.952 | **615.753** | **<0.001** | 113.957 | 3327.151 |
| **Gender (Ref**: Female**)** | | |  |  |  | |  |  |  |  |  |  |  |  |  |  |  |
| Male | **0.498** | **<0.001** | 0.425 | 0.584 | 0.757 | | 0.148 | 0.520 | 1.104 | 1.632 | 0.051 | 0.998 | 2.670 | 1.056 | 0.896 | 0.463 | 2.410 |
| **Age** (**Ref**: 15-29 years) | |  |  |  |  | |  |  |  |  |  |  |  |  |  |  |  |
| 30-44 | **0.618** | **<0.001** | 0.504 | 0.758 | 1.408 | | 0.143 | 0.891 | 2.225 | 1.148 | 0.559 | 0.723 | 1.823 | 1.300 | 0.722 | 0.307 | 5.506 |
| 45-59 | **0.583** | **<0.001** | 0.431 | 0.787 | 1.510 | | 0.217 | 0.785 | 2.903 | 1.279 | 0.563 | 0.555 | 2.949 | 0.634 | 0.575 | 0.129 | 3.122 |
| 60 and above | **0.643** | **0.026** | 0.436 | 0.949 | **3.718** | | **0.001** | 1.700 | 8.128 | 3.029 | 0.063 | 0.943 | 9.732 | 2.118 | 0.377 | 0.401 | 11.186 |
| **Educational attainment** (**Ref**: Primary school or below) | | | | | | |  |  |  |  |  |  |  |  |  |  |  |
| Junior high | 0.995 | 0.977 | 0.721 | 1.374 | 0.720 | | 0.367 | 0.352 | 1.471 | 0.994 | 0.990 | 0.399 | 2.480 | 0.875 | 0.844 | 0.231 | 3.307 |
| ≥Senior high | 1.107 | 0.531 | 0.806 | 1.521 | 0.861 | | 0.675 | 0.426 | 1.738 | 1.100 | 0.832 | 0.455 | 2.662 | 0.622 | 0.455 | 0.179 | 2.161 |
| **Marital status** (**Ref:** Single) | | | |  |  | |  |  |  |  |  |  |  |  |  |  |  |
| Married/ Cohabiting | **7.147** | **<0.001** | 5.113 | 9.991 | 0.876 | | 0.751 | 0.388 | 1.979 | 1.991 | 0.121 | 0.834 | 4.753 | <0.001 | ＞0.99 | <0.001 | ＞0.99 |
| Divorced/  Widowed | **5.876** | **<0.001** | 3.364 | 10.261 | 0.903 | | 0.869 | 0.269 | 3.031 | 9.056 | 0.057 | 0.940 | 87.243 | <0.001 | ＞0.99 | <0.001 | ＞0.99 |
| **Monthly per capita household income ranking** (**Ref:** Lowest) | | | | | |  |  |  |  |  |  |  |  |  |  |  |  |
| Lower | 1.085 | 0.575 | 0.815 | 1.445 | 0.801 | | 0.477 | 0.434 | 1.478 | 1.935 | 0.060 | 0.974 | 3.844 | **10.063** | **0.008** | 1.827 | 55.424 |
| Middle | 1.026 | 0.859 | 0.775 | 1.357 | 0.913 | | 0.768 | 0.500 | 1.669 | **2.154** | **0.029** | 1.080 | 4.292 | 2.961 | 0.162 | 0.646 | 13.566 |
| Higher | 1.098 | 0.512 | 0.831 | 1.449 | 1.426 | | 0.262 | 0.767 | 2.653 | 1.768 | 0.083 | 0.928 | 3.368 | 2.315 | 0.277 | 0.510 | 10.517 |
| Highest | 1.023 | 0.877 | 0.771 | 1.357 | 0.921 | | 0.788 | 0.505 | 1.681 | 1.652 | 0.139 | 0.850 | 3.210 | 3.502 | 0.103 | 0.776 | 15.811 |
| **Self-rating of health** (**Ref**: Poor) | | |  |  |  | |  |  |  |  |  |  |  |  |  |  |  |
| General | **0.465** | **<0.001** | 0.341 | 0.634 | 1.178 | | 0.552 | 0.686 | 2.026 | 1.127 | 0.794 | 0.459 | 2.764 | 0.416 | 0.067 | 0.162 | 1.064 |
| Good | **0.561** | **<0.001** | 0.408 | 0.769 | **2.460** | | **0.003** | 1.366 | 4.429 | 1.066 | 0.891 | 0.430 | 2.643 | 1.593 | 0.420 | 0.513 | 4.942 |
| **Distance of migration** (**Ref**: Inter-county) | | | |  |  | |  |  |  |  |  |  |  |  |  |  |  |
| Inter-city | 0.881 | 0.260 | 0.707 | 1.098 | 0.789 | | 0.311 | 0.499 | 1.248 | 0.758 | 0.290 | 0.453 | 1.267 | **0.309** | **0.013** | 0.122 | 0.782 |
| Inter-province | **0.501** | **<0.001** | 0.396 | 0.635 | 0.820 | | 0.442 | 0.494 | 1.360 | 1.010 | 0.974 | 0.562 | 1.814 | **0.154** | **0.001** | 0.050 | 0.468 |
| **Years of residing in migration destination** (**Ref**: <1) | | | | | | |  |  |  |  |  |  |  |  |  |  |  |
| 1-4 | 0.961 | 0.761 | 0.742 | 1.244 | **2.564** | | **<0.001** | 1.552 | 4.234 | 1.106 | 0.739 | 0.610 | 2.006 | 0.630 | 0.532 | 0.148 | 2.676 |
| 5-9 | 0.832 | 0.190 | 0.632 | 1.095 | **2.191** | | **0.004** | 1.278 | 3.755 | 1.580 | 0.184 | 0.805 | 3.104 | 0.663 | 0.589 | 0.150 | 2.940 |
| 10 and above | 0.908 | 0.518 | 0.678 | 1.216 | **2.304** | | **0.006** | 1.278 | 4.155 | 1.356 | 0.425 | 0.642 | 2.864 | 0.770 | 0.737 | 0.167 | 3.542 |
| **Migration destination** (**Ref**: Pearl River Delta) | | | | | | |  |  |  |  |  |  |  |  |  |  |  |
| Yangtze River delta | **1.525** | **0.010** | 1.104 | 2.106 | 0.839 | | 0.643 | 0.400 | 1.761 | 1.252 | 0.651 | 0.473 | 3.315 | <0.001 | >0.99 | <0.001 | >0.99 |
| Circum-Bohai Sea | 1.376 | 0.058 | 0.989 | 1.914 | 1.818 | | 0.143 | 0.818 | 4.040 | **0.329** | **0.014** | 0.135 | 0.802 | <0.001 | >0.99 | <0.001 | >0.99 |
| others | **1.715** | **<0.001** | 1.269 | 2.318 | 1.212 | | 0.596 | 0.594 | 2.474 | 0.882 | 0.780 | 0.364 | 2.136 | <0.001 | >0.99 | <0.001 | >0.99 |
| **R^2^** | 0.1017 | | | | 0.0960 | | | | | 0.0694 | | | | 0.6034 | | | |

# Table S7. Heckman two-step models on burdens of hospital care: subgroup analyses in study participants divided by household income (in quintiles)

**(a)**

| **Variable** | **Natural logarithm of total hospital expenditure (n=13065)** | | | | | | | | | | | | | | | | | | | |
| --- | --- | --- | --- | --- | --- | --- | --- | --- | --- | --- | --- | --- | --- | --- | --- | --- | --- | --- | --- | --- |
|  | **Lowest income (n=3611)** | | | | **Lower income (n=2713)** | | | | **Middle income (n=2459)** | | | | **Higher income (n=2194)** | | | | **Highest income (n=2088)** | | | |
|  | **Selection model (hospital admission)** | | **Outcome model** | | **Selection model (hospital admission)** | | **Outcome model** | | **Selection model (hospital admission)** | | **Outcome model** | | **Selection model (hospital admission)** | | **Outcome model** | | **Selection model (insurance reimbursement)** | | **Outcome model** | |
|  | **Coef** | **P** | **Coef** | **P** | **Coef** | **P** | **Coef** | **P** | **Coef** | **P** | **Coef** | **P** | **Coef** | **P** | **Coef** | **P** | **Coef** | **P** | **Coef** | **P** |
| **Type of health insurance** (**Ref:** BMIURR) | | | | | | | | | |  |  |  |  |  |  |  |  |  |  |  |
| BMIUE | -0.084 | 0.392 | 0.149 | 0.233 | 0.016 | 0.869 | **0.220** | **0.036** | -0.078 | 0.396 | 0.199 | 0.063 | 0.198 | 0.037 | 0.183 | 0.138 | -0.144 | 0.165 | -0.030 | 0.818 |
| Free medical care | -0.079 | 0.912 | **3.379** | **<0.001** | -0.482 | 0.373 | 0.837 | 0.310 | -0.126 | 0.836 | **1.915** | **0.019** | 0.071 | 0.841 | -0.543 | 0.219 | -0.575 | 0.161 | 0.131 | 0.835 |
| **Local fund** (**Ref**: no) | | | | | | | | | |  |  |  |  |  |  |  |  |  |  |  |
| yes | 0.443 | <0.001 | -0.081 | 0.440 | 0.485 | <0.001 | -0.049 | 0.709 | 0.547 | <0.001 | -0.316 | 0.108 | 0.406 | <0.001 | -0.173 | 0.208 | 0.544 | <0.001 | -0.154 | 0.496 |
| **Location of insurance settlement** (**Ref:** None-residential location) | | | | | | | | | |  |  |  |  |  |  |  |  |  |  |  |
| Residential location | |  | -0.123 | 0.166 |  |  | -0.109 | 0.280 |  |  | **-0.237** | **0.038** |  |  | 0.040 | 0.741 |  |  | -0.205 | -0.205 |
| **Gender** (**Ref**: Female) | | | | | | | | | |  |  |  |  |  |  |  |  |  |  |  |
| Male | -0.217 | <0.001 | 0.095 | 0.288 | -0.370 | <0.001 | 0.155 | 0.228 | -0.286 | <0.001 | 0.173 | 0.227 | -0.203 | 0.005 | 0.151 | 0.180 | -0.293 | <0.001 | **0.440** | **0.003** |
| **Age** (**Ref**: 15-29 years) | | | | | | | | | |  |  |  |  |  |  |  |  |  |  |  |
| 30-44 | -0.609 | <0.001 | -0.094 | 0.549 | -0.360 | <0.001 | -0.019 | 0.880 | -0.403 | <0.001 | 0.257 | 0.121 | -0.406 | <0.001 | -0.016 | 0.908 | -0.164 | 0.082 | 0.176 | 0.165 |
| 45-59 | -0.523 | <0.001 | 0.089 | 0.584 | -0.384 | <0.001 | -0.060 | 0.683 | -0.427 | <0.001 | 0.151 | 0.455 | -0.444 | <0.001 | 0.261 | 0.155 | 0.018 | 0.894 | 0.144 | 0.422 |
| 60 and above | -0.349 | 0.002 | -0.005 | 0.975 | -0.007 | 0.963 | 0.012 | 0.941 | <0.001 | 0.998 | -0.102 | 0.602 | 0.008 | 0.962 | 0.074 | 0.709 | 0.221 | 0.204 | **0.515** | **0.032** |
| **Educational attainment** (**Ref:** Primary school or below) | | | | | | | | | |  |  |  |  |  |  |  |  |  |  |  |
| Junior high | 0.103 | 0.099 | 0.140 | 0.123 | 0.089 | 0.279 | 0.125 | 0.251 | 0.163 | 0.093 | 0.127 | 0.387 | 0.009 | 0.945 | 0.123 | 0.443 | 0.235 | 0.137 | 0.207 | 0.375 |
| ≥Senior high | 0.291 | <0.001 | 0.068 | 0.590 | 0.308 | 0.001 | 0.190 | 0.166 | 0.279 | 0.008 | 0.175 | 0.311 | 0.092 | 0.464 | 0.202 | 0.214 | 0.290 | 0.070 | 0.043 | 0.854 |
| **Marital status** (**Ref:** Single) | | | | | | | | | |  |  |  |  |  |  |  |  |  |  |  |
| Married/ Cohabiting | 0.982 | <0.001 | 0.270 | 0.487 | 0.578 | 0.003 | -0.144 | 0.611 | 0.938 | <0.001 | -0.098 | 0.814 | 1.232 | <0.001 | 0.050 | 0.889 | 0.898 | <0.001 | -0.042 | 0.906 |
| Divorced/ Widowed | 0.834 | <0.001 | -0.114 | 0.783 | 0.338 | 0.203 | -0.693 | 0.056 | 0.580 | 0.017 | 0.002 | 0.997 | 1.060 | <0.001 | -0.131 | 0.748 | 0.740 | <0.001 | -0.089 | 0.807 |
| **Self-rated health** (**Ref**: Poor) | | | | | | | | | |  |  |  |  |  |  |  |  |  |  |  |
| General | -0.390 | <0.001 | -0.102 | 0.422 | -0.074 | 0.479 | **-0.364** | **0.006** | -0.302 | 0.010 | -0.518 | 0.006 | -0.689 | <0.001 | -0.377 | 0.066 | -0.444 | 0.007 | 0.326 | 0.205 |
| Good | -0.314 | <0.001 | **-0.390** | **0.001** | 0.077 | 0.466 | **-0.603** | **<0.001** | -0.189 | 0.109 | **-0.639** | **<0.001** | -0.674 | <0.001 | **-0.551** | **0.007** | -0.454 | 0.007 | 0.218 | 0.409 |
| **Distance of migration** (**Ref**: Inter-county) | | | | | | | | | |  |  |  |  |  |  |  |  |  |  |  |
| Inter-city | -0.258 | <0.001 |  |  | -0.282 | 0.001 |  |  | -0.135 | 0.125 |  |  | -0.116 | 0.207 |  |  | 0.074 | 0.506 |  |  |
| Inter-province | -0.473 | <0.001 |  |  | -0.455 | <0.001 |  |  | -0.335 | <0.001 |  |  | -0.568 | <0.001 |  |  | -0.247 | 0.032 |  |  |
| **Years of residing in migration destination** (**Ref**: <1) | | | | | | | | | |  |  |  |  |  |  |  |  |  |  |  |
| 1-4 | 0.211 | 0.040 |  |  | 0.103 | 0.349 |  |  | 0.032 | 0.770 |  |  | 0.082 | 0.460 |  |  | 0.074 | 0.521 |  |  |
| 5-9 | 0.262 | 0.011 |  |  | 0.066 | 0.567 |  |  | 0.022 | 0.852 |  |  | 0.041 | 0.740 |  |  | 0.015 | 0.909 |  |  |
| 10 and above | 0.136 | 0.192 |  |  | 0.091 | 0.448 |  |  | 0.095 | 0.432 |  |  | 0.074 | 0.567 |  |  | 0.174 | 0.209 |  |  |
| **Migration destination** (**Ref**: Pearl River Delta) | | | | | | | | | |  |  |  |  |  |  |  |  |  |  |  |
| Yangtze River delta | -0.064 | 0.650 | 0.217 | 0.302 | -0.047 | 0.738 | 0.279 | 0.131 | 0.118 | 0.395 | -0.010 | 0.959 | -0.216 | 0.188 | 0.153 | 0.498 | 0.174 | 0.321 | 0.451 | 0.096 |
| Circum-Bohai Sea | -0.006 | 0.968 | 0.082 | 0.694 | 0.118 | 0.408 | 0.353 | 0.058 | 0.288 | 0.050 | 0.090 | 0.677 | -0.132 | 0.423 | -0.008 | 0.972 | 0.060 | 0.739 | 0.365 | 0.162 |
| others | 0.117 | 0.351 | -0.286 | 0.131 | 0.179 | 0.139 | -0.016 | 0.926 | 0.337 | 0.007 | -0.093 | 0.684 | 0.040 | 0.784 | 0.006 | 0.975 | 0.309 | 0.054 | 0.034 | 0.903 |
| **mills** | **Coef** | | **P** | | **Coef** | | **P** | | **Coef** | | **P** | | **Coef** | | **P** | | **Coef** | | **P** | |
| $\boldsymbol{\lambda}_{\boldsymbol{i}}$ | -0.140 | | 0.585 | | 0.206 | | 0.497 | | -0.096 | | 0.834 | | 0.202 | | 0.473 | | -0.547 | | 0.197 | |

**(b)**

| **Variable** | **Natural logarithm of out-of-pocket payments (n=13065)** | | | | | | | | | | | | | | | | | | | |
| --- | --- | --- | --- | --- | --- | --- | --- | --- | --- | --- | --- | --- | --- | --- | --- | --- | --- | --- | --- | --- |
|  | **Lowest income (n=3611)** | | | | **Lower income (n=2713)** | | | | **Middle income (n=2459)** | | | | **Higher income (n=2194)** | | | | **Highest income (n=2088)** | | | |
|  | **Selection model (hospital admission)** | | **Outcome model** | | **Selection model (hospital admission)** | | **Outcome model** | | **Selection model (hospital admission)** | | **Outcome model** | | **Selection model (hospital admission)** | | **Outcome model** | | **Selection model (insurance reimbursement)** | | **Outcome model** | |
|  | **Coef** | **P** | **Coef** | **P** | **Coef** | **P** | **Coef** | **P** | **Coef** | **P** | **Coef** | **P** | **Coef** | **P** | **Coef** | **P** | **Coef** | **P** | **Coef** | **P** |
| **Type of health insurance** (**Ref:** BMIURR) | | | | | | |  |  |  |  |  |  |  |  |  |  |  |  |  |  |
| BMIUE | -0.084 | 0.392 | -0.152 | 0.304 | 0.016 | 0.869 | -0.113 | 0.404 | -0.078 | 0.396 | -0.073 | 0.565 | 0.198 | 0.037 | 0.059 | 0.699 | -0.144 | 0.165 | **-0.426** | **0.009** |
| Free medical care | -0.079 | 0.912 | **2.844** | **0.007** | -0.482 | 0.373 | 0.669 | 0.532 | -0.126 | 0.836 | 1.789 | 0.064 | 0.071 | 0.841 | -0.858 | 0.118 | -0.575 | 0.161 | -0.182 | 0.817 |
| **Local fund** (**Ref**: no) | | | | | | |  |  |  |  |  |  |  |  |  |  |  |  |  |  |
| yes | 0.443 | <0.001 | -0.187 | 0.132 | 0.485 | <0.001 | -0.039 | 0.819 | 0.547 | <0.001 | **-0.542** | **0.020** | 0.406 | <0.001 | -0.322 | 0.059 | 0.544 | <0.001 | -0.248 | 0.379 |
| **Location of insurance settlement** (**Ref:** None-residential location) | | | | | | |  |  |  |  |  |  |  |  |  |  |  |  |  |  |
| Residential location | |  | **-0.241** | **0.022** |  |  | -0.190 | 0.145 |  |  | **-0.493** | **<0.001** |  |  | 0.076 | 0.609 |  |  | **-0.416** | **0.028** |
| **Gender** (**Ref**: Female) | | | | | | |  |  |  |  |  |  |  |  |  |  |  |  |  |  |
| Male | -0.217 | <0.001 | -0.002 | 0.981 | -0.370 | <0.001 | 0.160 | 0.339 | -0.286 | <0.001 | 0.076 | 0.651 | -0.203 | 0.005 | -0.031 | 0.824 | -0.293 | <0.001 | **0.499** | **0.008** |
| **Age** (**Ref**: 15-29 years) | | | | | | |  |  |  |  |  |  |  |  |  |  |  |  |  |  |
| 30-44 | -0.609 | <0.001 | -0.217 | 0.242 | -0.360 | <0.001 | -0.172 | 0.279 | -0.403 | <0.001 | 0.362 | 0.065 | -0.406 | <0.001 | -0.149 | 0.391 | -0.164 | 0.082 | 0.052 | 0.743 |
| 45-59 | -0.523 | <0.001 | -0.063 | 0.744 | -0.384 | <0.001 | -0.120 | 0.532 | -0.427 | <0.001 | 0.023 | 0.923 | -0.444 | <0.001 | 0.231 | 0.309 | 0.018 | 0.894 | -0.309 | 0.168 |
| 60 and above | -0.349 | 0.002 | -0.149 | 0.435 | -0.007 | 0.963 | -0.094 | 0.666 | 0.000 | 0.998 | -0.279 | 0.225 | 0.008 | 0.962 | -0.102 | 0.678 | 0.221 | 0.204 | 0.177 | 0.554 |
| **Educational attainment** (**Ref:** Primary school or below) | | | | | | |  |  |  |  |  |  |  |  |  |  |  |  |  |  |
| Junior high | 0.103 | 0.099 | 0.203 | 0.059 | 0.089 | 0.279 | 0.154 | 0.274 | 0.163 | 0.093 | 0.165 | 0.338 | 0.009 | 0.945 | 0.061 | 0.758 | 0.235 | 0.137 | -0.083 | 0.776 |
| ≥Senior high | 0.291 | <0.001 | 0.209 | 0.160 | 0.308 | 0.001 | 0.238 | 0.181 | 0.279 | 0.008 | 0.058 | 0.775 | 0.092 | 0.464 | 0.205 | 0.311 | 0.290 | 0.070 | -0.149 | 0.612 |
| **Marital status** (**Ref:** Single) | | | | | | |  |  |  |  |  |  |  |  |  |  |  |  |  |  |
| Married/ Cohabiting | 0.982 | <0.001 | 0.800 | 0.083 | 0.578 | 0.003 | 0.058 | 0.874 | 0.938 | <0.001 | -0.264 | 0.588 | 1.232 | <0.001 | 0.338 | 0.442 | 0.898 | <0.001 | 0.115 | 0.795 |
| Divorced/ Widowed | 0.834 | 0.001 | 0.319 | 0.515 | 0.338 | 0.203 | -0.611 | 0.194 | 0.580 | 0.017 | -0.221 | 0.652 | 1.060 | <0.001 | -0.082 | 0.870 | 0.740 | <0.001 | 0.137 | 0.763 |
| **Self-rated health** (**Ref**: Poor) | | | | | | |  |  |  |  |  |  |  |  |  |  |  |  |  |  |
| General | -0.390 | <0.001 | -0.111 | 0.458 | -0.074 | 0.479 | -0.268 | 0.119 | -0.302 | 0.010 | **-0.622** | **0.006** | -0.689 | <0.001 | **-0.555** | **0.030** | -0.444 | 0.007 | 0.315 | 0.325 |
| Good | -0.314 | <0.001 | **-0.458** | **0.002** | 0.077 | 0.466 | **-0.493** | **0.006** | -0.189 | 0.109 | **-0.752** | **<0.001** | -0.674 | <0.001 | **-0.609** | **0.016** | -0.454 | 0.007 | 0.208 | 0.528 |
| **Distance of migration** (**Ref**: Inter-county) | | | | | | |  |  |  |  |  |  |  |  |  |  |  |  |  |  |
| Inter-city | -0.258 | <0.001 |  |  | -0.282 | 0.001 |  |  | -0.135 | 0.125 |  |  | -0.116 | 0.207 |  |  | 0.074 | 0.506 |  |  |
| Inter-province | -0.473 | <0.001 |  |  | -0.455 | <0.001 |  |  | -0.335 | <0.001 |  |  | -0.568 | <0.001 |  |  | -0.247 | 0.032 |  |  |
| **Years of residing in migration destination** (**Ref**: <1) | | | | | | |  |  |  |  |  |  |  |  |  |  |  |  |  |  |
| 1-4 | 0.211 | 0.040 |  |  | 0.103 | 0.349 |  |  | 0.032 | 0.770 |  |  | 0.082 | 0.460 |  |  | 0.074 | 0.521 |  |  |
| 5-9 | 0.262 | 0.011 |  |  | 0.066 | 0.567 |  |  | 0.022 | 0.852 |  |  | 0.041 | 0.740 |  |  | 0.015 | 0.909 |  |  |
| 10 and above | 0.136 | 0.192 |  |  | 0.091 | 0.448 |  |  | 0.095 | 0.432 |  |  | 0.074 | 0.567 |  |  | 0.174 | 0.209 |  |  |
| **Migration destination** (**Ref**: Pearl River Delta) | | | | | | |  |  |  |  |  |  |  |  |  |  |  |  |  |  |
| Yangtze River delta | -0.064 | 0.650 | 0.448 | 0.073 | -0.047 | 0.738 | 0.468 | 0.051 | 0.118 | 0.395 | 0.479 | 0.041 | -0.216 | 0.188 | 0.250 | 0.370 | 0.174 | 0.321 | 0.898 | 0.008 |
| Circum-Bohai Sea | -0.006 | 0.968 | 0.317 | 0.196 | 0.118 | 0.408 | 0.342 | 0.158 | 0.288 | 0.050 | 0.595 | 0.020 | -0.132 | 0.423 | 0.185 | 0.481 | 0.060 | 0.739 | 0.779 | 0.017 |
| others | 0.117 | 0.351 | -0.089 | 0.691 | 0.179 | 0.139 | 0.083 | 0.706 | 0.337 | 0.007 | 0.352 | 0.190 | 0.040 | 0.784 | 0.289 | 0.219 | 0.309 | 0.054 | 0.421 | 0.231 |
| **mills** | **Coef** | | **P** | | **Coef** | | **P** | | **Coef** | | **P** | | **Coef** | | **P** | | **Coef** | | **P** | |
| $\boldsymbol{\lambda}_{\boldsymbol{i}}$ | 0.009 | | 0.978 | | 0.216 | | 0.581 | | -0.320 | | 0.554 | | 0.539 | | 0.121 | | -0.608 | | 0.250 | |

**(c)**

| **Variable** | **Reimbursement ratio of hospital expenditure (n=3186)** | | | | | | | | | | | | | | | | | | | |
| --- | --- | --- | --- | --- | --- | --- | --- | --- | --- | --- | --- | --- | --- | --- | --- | --- | --- | --- | --- | --- |
|  | **Lowest income (n=939)** | | | | **Lower income (n=662)** | | | | **Middle income (n=574)** | | | | **Higher income (n=544)** | | | | **Highest income (n=467)** | | | |
|  | **Selection model (hospital admission)** | | **Outcome model** | | **Selection model (hospital admission)** | | **Outcome model** | | **Selection model (hospital admission)** | | **Outcome model** | | **Selection model (hospital admission)** | | **Outcome model** | | **Selection model (insurance reimbursement)** | | **Outcome model** | |
|  | **Coef** | **P** | **Coef** | **P** | **Coef** | **P** | **Coef** | **P** | **Coef** | **P** | **Coef** | **P** | **Coef** | **P** | **Coef** | **P** | **Coef** | **P** | **Coef** | **P** |
| **Type of health insurance** (**Ref:** BMIURR) | | | | |  |  |  |  |  |  |  |  |  |  |  |  |  |  |  |  |
| BMIUE | 0.145 | 0.415 | **0.124** | **<0.001** | 0.441 | 0.014 | **0.189** | **0.002** | 0.512 | 0.006 | **0.121** | **0.002** | 0.173 | 0.358 | **0.117** | **<0.001** | 0.134 | 0.493 | **0.198** | **<0.001** |
| Free medical care | 4.798 | >0.99 | 0.205 | 0.382 | 5.460 | >0.99 | 0.215 | 0.469 | 5.285 | >0.99 | 0.091 | 0.671 | 5.406 | >0.99 | 0.232 | 0.062 | 5.116 | >0.99 | **0.371** | **0.007** |
| **Local fund** (**Ref**: no) | | | | |  |  |  |  |  |  |  |  |  |  |  |  |  |  |  |  |
| yes | 0.482 | <0.001 | 0.038 | 0.369 | 0.522 | <0.001 | 0.081 | 0.260 | 0.627 | <0.001 | 0.001 | 0.980 | 0.744 | <0.001 | **0.126** | **0.014** | 0.826 | <0.001 | -0.045 | 0.533 |
| **Location of insurance settlement** (**Ref:** None-residential location) | | | | | | |  |  |  |  |  |  |  |  |  |  |  |  |  |  |
| Residential location | | | **0.053** | **0.015** |  |  | **0.046** | **0.080** |  |  | **0.145** | **<0.001** |  |  | 0.005 | 0.886 |  |  | 0.058 | 0.136 |
| **Gender** (**Ref**: Female) | | | | |  |  |  |  |  |  |  |  |  |  |  |  |  |  |  |  |
| Male | -0.118 | 0.263 | **0.058** | **0.008** | -0.075 | 0.568 | 0.007 | 0.809 | -0.035 | 0.806 | 0.035 | 0.146 | 0.422 | 0.007 | 0.061 | 0.073 | 0.031 | 0.848 | -0.014 | 0.646 |
| **Age** (**Ref**: 15-29 years) | | | | |  |  |  |  |  |  |  |  |  |  |  |  |  |  |  |  |
| 30-44 | 0.032 | 0.812 | 0.041 | 0.155 | 0.037 | 0.794 | 0.044 | 0.160 | 0.015 | 0.921 | 0.013 | 0.624 | 0.184 | 0.247 | 0.021 | 0.486 | 0.272 | 0.107 | 0.013 | 0.752 |
| 45-59 | 0.338 | 0.042 | 0.038 | 0.360 | 0.329 | 0.077 | 0.072 | 0.228 | 0.436 | 0.046 | 0.065 | 0.135 | 0.249 | 0.308 | -0.032 | 0.478 | 0.629 | 0.021 | 0.076 | 0.241 |
| 60 and above | 0.396 | 0.051 | 0.032 | 0.504 | 0.908 | 0.003 | 0.167 | 0.144 | 0.264 | 0.376 | 0.045 | 0.385 | 1.005 | 0.004 | 0.082 | 0.231 | 0.528 | 0.122 | 0.031 | 0.684 |
| **Educational attainment** (**Ref:** Primary school or below) | | | | | | |  |  |  |  |  |  |  |  |  |  |  |  |  |  |
| Junior high | 0.157 | 0.167 | **-0.050** | **0.042** | 0.017 | 0.913 | -0.009 | 0.780 | 0.140 | 0.476 | -0.010 | 0.762 | 0.076 | 0.749 | 0.013 | 0.772 | 0.184 | 0.558 | 0.038 | 0.526 |
| ≥Senior high | 0.185 | 0.181 | **-0.059** | **0.049** | 0.169 | 0.316 | <0.001 | 0.997 | 0.208 | 0.321 | 0.034 | 0.378 | 0.268 | 0.268 | -0.005 | 0.910 | 0.350 | 0.268 | -0.009 | 0.882 |
| **Marital status** (**Ref:** Single) | | | | |  |  |  |  |  |  |  |  |  |  |  |  |  |  |  |  |
| Married/ Cohabiting | 0.102 | 0.813 | **-0.191** | **0.029** | 0.028 | 0.941 | -0.063 | 0.450 | -0.698 | 0.090 | -0.007 | 0.924 | 0.580 | 0.036 | -0.026 | 0.716 | 0.024 | 0.916 | -0.070 | 0.137 |
| Divorced/ Widowed | 0.176 | 0.723 | -0.177 | 0.066 | -0.190 | 0.711 | -0.041 | 0.719 | -0.480 | 0.432 | 0.074 | 0.443 | 1.490 | 0.027 | 0.083 | 0.454 | 0.310 | 0.528 | -0.059 | 0.463 |
| **Self-rated health** (**Ref**: Poor) | | | | |  |  |  |  |  |  |  |  |  |  |  |  |  |  |  |  |
| General | -0.133 | 0.324 | -0.008 | 0.751 | 0.089 | 0.669 | 0.035 | 0.422 | -0.158 | 0.517 | 0.021 | 0.581 | -0.126 | 0.595 | 0.014 | 0.726 | -0.458 | 0.203 | 0.045 | 0.461 |
| Good | -0.456 | 0.001 | 0.031 | 0.476 | -0.060 | 0.771 | 0.017 | 0.697 | -0.300 | 0.220 | 0.033 | 0.422 | -0.127 | 0.592 | -0.037 | 0.378 | -0.598 | 0.099 | 0.051 | 0.448 |
| **Distance of migration** (**Ref**: Inter-county) | | | | |  |  |  |  |  |  |  |  |  |  |  |  |  |  |  |  |
| Inter-city | -0.325 | 0.007 | 0.003 | 0.934 | -0.152 | 0.296 | -0.050 | 0.157 | -0.342 | 0.044 | 0.048 | 0.151 | -0.349 | 0.039 | -0.035 | 0.309 | 0.225 | 0.249 | -0.034 | 0.415 |
| Inter-province | -0.723 | <0.001 | 0.023 | 0.697 | -0.390 | 0.010 | -0.072 | 0.231 | -0.330 | 0.062 | 0.042 | 0.215 | -0.652 | 0.001 | -0.087 | 0.069 | -0.192 | 0.344 | 0.008 | 0.857 |
| **Years of residing in migration destination** (**Ref**: <1) | | | | | | |  |  |  |  |  |  |  |  |  |  |  |  |  |  |
| 1-4 | 0.065 | 0.703 |  |  | -0.088 | 0.640 |  |  | -0.162 | 0.437 |  |  | -0.259 | 0.214 |  |  | 0.272 | 0.166 |  |  |
| 5-9 | 0.203 | 0.246 |  |  | -0.014 | 0.944 |  |  | -0.344 | 0.112 |  |  | 0.040 | 0.868 |  |  | 0.517 | 0.029 |  |  |
| 10 and above | 0.045 | 0.807 |  |  | -0.010 | 0.963 |  |  | -0.064 | 0.792 |  |  | -0.378 | 0.140 |  |  | 0.345 | 0.183 |  |  |
| **Migration destination** (**Ref**: Pearl River Delta) | | | | |  |  |  |  |  |  |  |  |  |  |  |  |  |  |  |  |
| Yangtze River delta | -0.349 | 0.134 | -0.011 | 0.852 | -0.351 | 0.173 | -0.090 | 0.223 | -0.197 | 0.490 | -0.128 | 0.011 | -0.438 | 0.185 | -0.042 | 0.515 | -0.028 | 0.939 | -0.125 | 0.072 |
| Circum-Bohai Sea | -0.502 | 0.035 | -0.017 | 0.792 | -0.367 | 0.154 | -0.038 | 0.616 | -0.398 | 0.159 | -0.162 | 0.004 | -0.776 | 0.018 | -0.119 | 0.102 | -0.088 | 0.809 | -0.131 | 0.062 |
| others | -0.094 | 0.663 | -0.021 | 0.667 | -0.032 | 0.889 | -0.038 | 0.438 | 0.143 | 0.573 | -0.149 | 0.001 | -0.196 | 0.520 | -0.088 | 0.095 | -0.178 | 0.576 | -0.121 | 0.063 |
| **mills** | **Coef** | | **P** | | **Coef** | | **P** | | **Coef** | | **P** | | **Coef** | | **P** | | **Coef** | | **P** | |
| $\boldsymbol{\lambda}_{\boldsymbol{i}}$ | -0.132 | | 0.412 | | 0.227 | | 0.385 | | -0.097 | | 0.477 | | 0.082 | | 0.485 | | -0.207 | | 0.184 | |

# Table S8. Heckman two-step models on burdens of hospital care in BMIURR enrollees

| **Variable** | **Natural logarithm of total hospital expenditure (n=9707)** | | | | **Natural logarithm of out-of-pocket payments (n=9707)** | | | | | **Reimbursement ratio of hospital expenditure (n=2290)** | | | |
| --- | --- | --- | --- | --- | --- | --- | --- | --- | --- | --- | --- | --- | --- |
|  | **Selection model (hospital admission)** | | **Outcome model** | | **Selection model (hospital admission)** | | **Outcome model** | | | **Selection model (insurance reimbursement)** | | **Outcome model** | |
|  | **Coef** | **P** | **Coef** | **P** | **Coef** | **P** | **Coef** | | **P** | **Coef** | **P** | **Coef** | **P** |
| **Local fund** (**Ref**: no) | | | | | | | | | | | | | |
| yes | 0.575 | <0.001 | -0.127 | 0.125 | 0.575 | <0.001 | | **-0.287** | **0.004** | 0.696 | <0.001 | **0.099** | **0.036** |
| **Location of insurance settlement** (**Ref:** None-residential location) | | | | | | | | | | | | | |
| Residential location | | | **-0.124** | **0.028** |  |  | | **-0.262** | **<0.001** |  |  | **0.065** | **<0.001** |
| **Gender** (**Ref**: Female) | | | | | | | | | | | | | |
| Male | -0.219 | <0.001 | **0.176** | **0.004** | -0.219 | <0.001 | | **0.144** | **0.050** | -0.045 | 0.496 | **0.029** | **0.037** |
| **Age** (**Ref**: 15-29 years) | | | | | | | | | | | | | |
| 30-44 | -0.469 | <0.001 | 0.046 | 0.601 | -0.469 | <0.001 | | 0.010 | 0.923 | 0.049 | 0.514 | **0.037** | **0.032** |
| 45-59 | -0.453 | <0.001 | 0.069 | 0.482 | -0.453 | <0.001 | | 0.005 | 0.967 | 0.351 | <0.001 | 0.062 | 0.054 |
| 60 and above | -0.246 | 0.001 | 0.007 | 0.949 | -0.246 | 0.001 | | -0.089 | 0.472 | 0.398 | 0.003 | **0.083** | **0.028** |
| **Educational attainment** (**Ref:** Primary school or below) | | | | | | | | | | | | | |
| Junior high | 0.110 | 0.011 | **0.142** | **0.027** | 0.110 | 0.011 | | **0.164** | **0.034** | 0.082 | 0.303 | -0.016 | 0.335 |
| ≥Senior high | 0.279 | <0.001 | 0.126 | 0.128 | 0.279 | <0.001 | | **0.195** | **0.049** | 0.193 | 0.030 | -0.025 | 0.272 |
| **Marital status** (**Ref:** Single) | | | | | | | | | | | | | |
| Married/ Cohabiting | 0.969 | <0.001 | 0.066 | 0.735 | 0.969 | <0.001 | | 0.136 | 0.560 | 0.022 | 0.889 | **-0.079** | **0.026** |
| Divorced/ Widowed | 0.738 | <0.001 | -0.211 | 0.315 | 0.738 | <0.001 | | -0.217 | 0.387 | 0.075 | 0.747 | -0.050 | 0.285 |
| **Monthly per capita household income ranking** (**Ref:** Lowest) | | | | | | | | | | | | | |
| Lower | -0.090 | 0.046 | 0.049 | 0.451 | -0.090 | 0.046 | | 0.015 | 0.842 | -0.053 | 0.496 | 0.006 | 0.723 |
| Middle | -0.038 | 0.441 | 0.106 | 0.117 | -0.038 | 0.441 | | 0.089 | 0.272 | 0.089 | 0.294 | 0.005 | 0.777 |
| Higher | -0.113 | 0.039 | **0.162** | **0.035** | -0.113 | 0.039 | | 0.133 | 0.149 | -0.036 | 0.701 | 0.010 | 0.611 |
| Highest | -0.003 | 0.961 | **0.249** | **0.003** | -0.003 | 0.961 | | **0.258** | **0.010** | -0.033 | 0.744 | -0.027 | 0.217 |
| **Self-rated health** (**Ref**: Poor) | | | | | | | | | | | | | |
| General | -0.316 | <0.001 | **-0.299** | **<0.001** | -0.316 | <0.001 | | **-0.282** | **0.005** | -0.118 | 0.219 | -0.005 | 0.790 |
| Good | -0.260 | <0.001 | **-0.522** | **<0.001** | -0.260 | <0.001 | | **-0.496** | **<0.001** | -0.307 | 0.001 | -0.016 | 0.571 |
| **Distance of migration** (**Ref**: Inter-county) | | | | | | | | | | | | | |
| Inter-city | -0.208 | <0.001 |  |  | -0.208 | <0.001 | |  |  | -0.225 | 0.003 | -0.021 | 0.307 |
| Inter-province | -0.453 | <0.001 |  |  | -0.453 | <0.001 | |  |  | -0.617 | <0.001 | -0.038 | 0.409 |
| **Years of residing in migration destination** (**Ref**: <1) | | | | | | | | | | | | | |
| 1-4 | 0.107 | 0.064 |  |  | 0.107 | 0.064 | |  |  | -0.041 | 0.673 |  |  |
| 5-9 | 0.142 | 0.019 |  |  | 0.142 | 0.019 | |  |  | 0.033 | 0.754 |  |  |
| 10 and above | 0.101 | 0.106 |  |  | 0.101 | 0.106 | |  |  | -0.050 | 0.655 |  |  |
| **Migration destination** (**Ref**: Pearl River Delta) | | | | | | | | | | | | | |
| Yangtze River delta | -0.116 | 0.179 | 0.188 | 0.156 | -0.116 | 0.179 | | **0.475** | **0.003** | -0.350 | 0.015 | **-0.113** | **0.014** |
| Circum-Bohai Sea | 0.032 | 0.712 | 0.111 | 0.386 | 0.032 | 0.712 | | **0.326** | **0.033** | -0.306 | 0.038 | **-0.090** | **0.025** |
| others | 0.122 | 0.101 | -0.157 | 0.165 | 0.122 | 0.101 | | 0.094 | 0.491 | -0.042 | 0.746 | **-0.080** | **0.005** |
| **mills** | **Coef** | | **P** | | **Coef** | | **P** | | | **Coef** | | **P** | |
| $\boldsymbol{\lambda}_{\boldsymbol{i}}$ | 0.031 | | 0.867 | | 0.002 | | 0.994 | | | 0.106 | | 0.456 | |

# Table S9. Heckman two-step models on burdens of hospital care in BMIUE enrollees

| **Variable** | **Natural logarithm of total hospital expenditure (n=3297)** | | | | **Natural logarithm of out-of-pocket payments (n=3297)** | | | | | **Reimbursement ratio of hospital expenditure (n=885)** | | | |
| --- | --- | --- | --- | --- | --- | --- | --- | --- | --- | --- | --- | --- | --- |
|  | **Selection model (hospital admission)** | | **Outcome model** | | **Selection model (hospital admission)** | | **Outcome model** | | | **Selection model (insurance reimbursement)** | | **Outcome model** | |
|  | **Coef** | **P** | **Coef** | **P** | **Coef** | **P** | **Coef** | | **P** | **Coef** | **P** | **Coef** | **P** |
| **Local fund** (**Ref**: no) | | | | | | | | | | | | | |
| yes | 0.160 | 0.080 | -0.004 | 0.973 | 0.160 | 0.080 | | 0.073 | 0.644 | 0.252 | 0.170 | -0.078 | 0.353 |
| **Location of insurance settlement** (**Ref:** None-residential location) | | | | | | | | | | | | | |
| Residential location | | | -0.098 | 0.432 |  |  | | -0.275 | 0.086 |  |  | 0.079 | 0.269 |
| **Gender** (**Ref**: Female) | | | | | | | | | | | | | |
| Male | -0.385 | <0.001 | 0.020 | 0.850 | -0.385 | <0.001 | | -0.222 | 0.118 | 0.235 | 0.078 | 0.004 | 0.943 |
| **Age** (**Ref**: 15-29 years) | | | | | | | | | | | | | |
| 30-44 | -0.231 | 0.001 | -0.024 | 0.787 | -0.231 | 0.001 | | -0.200 | 0.089 | 0.069 | 0.607 | 0.006 | 0.902 |
| 45-59 | -0.255 | 0.013 | 0.231 | 0.068 | -0.255 | 0.013 | | -0.059 | 0.725 | 0.126 | 0.587 | 0.029 | 0.723 |
| 60 and above | 0.064 | 0.640 | **0.318** | **0.039** | 0.064 | 0.640 | | 0.136 | 0.504 | 0.579 | 0.064 | -0.095 | 0.483 |
| **Educational attainment** (**Ref:** Primary school or below) | | | | | | | | | | | | | |
| Junior high | -0.047 | 0.665 | 0.184 | 0.142 | -0.047 | 0.665 | | 0.165 | 0.321 | -0.004 | 0.988 | -0.003 | 0.966 |
| ≥Senior high | 0.035 | 0.741 | **0.312** | **0.010** | 0.035 | 0.741 | | 0.206 | 0.200 | 0.043 | 0.855 | 0.014 | 0.854 |
| **Marital status** (**Ref:** Single) | | | | | | | | | | | | | |
| Married/ Cohabiting | 1.040 | <0.001 | 0.502 | 0.064 | 1.040 | <0.001 | | **0.985** | **0.006** | 0.363 | 0.157 | -0.120 | 0.292 |
| Divorced/ Widowed | 1.017 | <0.001 | 0.269 | 0.385 | 1.017 | <0.001 | | 0.688 | 0.092 | 1.154 | 0.040 | -0.155 | 0.433 |
| **Monthly per capita household income ranking** (**Ref:** Lowest) | | | | | | | | | | | | | |
| Lower | 0.098 | 0.321 | 0.108 | 0.356 | 0.098 | 0.321 | | 0.168 | 0.278 | 0.371 | 0.058 | -0.079 | 0.386 |
| Middle | 0.065 | 0.502 | -0.029 | 0.801 | 0.065 | 0.502 | | -0.058 | 0.700 | 0.418 | 0.031 | -0.051 | 0.592 |
| Higher | 0.146 | 0.129 | 0.084 | 0.462 | 0.146 | 0.129 | | 0.197 | 0.195 | 0.340 | 0.068 | -0.070 | 0.406 |
| Highest | 0.055 | 0.570 | 0.027 | 0.812 | 0.055 | 0.570 | | 0.020 | 0.896 | 0.290 | 0.128 | -0.036 | 0.658 |
| **Self-rated health** (**Ref**: Poor) | | | | | | | | | | | | | |
| General | -0.429 | <0.001 | -0.256 | 0.085 | -0.429 | <0.001 | | **-0.427** | **0.031** | 0.033 | 0.893 | 0.041 | 0.599 |
| Good | -0.289 | 0.009 | **-0.402** | **0.003** | -0.289 | 0.009 | | **-0.584** | **0.001** | 0.011 | 0.966 | 0.047 | 0.556 |
| **Distance of migration** (**Ref**: Inter-county) | | | | | | | | | | | | | |
| Inter-city | -0.128 | 0.093 |  |  | -0.128 | 0.093 | |  |  | -0.150 | 0.302 | 0.015 | 0.783 |
| Inter-province | -0.412 | <0.001 |  |  | -0.412 | <0.001 | |  |  | -0.015 | 0.925 | -0.047 | 0.409 |
| **Years of residing in migration destination** (**Ref**: <1) | | | | | | | | | | | | | |
| 1-4 | 0.095 | 0.305 |  |  | 0.095 | 0.305 | |  |  | 0.061 | 0.726 |  |  |
| 5-9 | 0.016 | 0.866 |  |  | 0.016 | 0.866 | |  |  | 0.269 | 0.164 |  |  |
| 10 and above | 0.076 | 0.462 |  |  | 0.076 | 0.462 | |  |  | 0.182 | 0.389 |  |  |
| **Migration destination** (**Ref**: Pearl River Delta) | | | | | | | | | | | | | |
| Yangtze River delta | 0.183 | 0.081 | **0.342** | **0.014** | 0.183 | 0.081 | | **0.731** | **<0.001** | 0.106 | 0.686 | -0.084 | 0.322 |
| Circum-Bohai Sea | 0.120 | 0.269 | **0.282** | **0.042** | 0.120 | 0.269 | | **0.671** | **<0.001** | -0.611 | 0.013 | 0.021 | 0.875 |
| others | 0.267 | 0.006 | 0.161 | 0.277 | 0.267 | 0.006 | | **0.571** | **0.004** | -0.070 | 0.771 | -0.077 | 0.347 |
| **mills** | **Coef** | | **P** | | **Coef** | | **P** | | | **Coef** | | **P** | |
| $\boldsymbol{\lambda}_{\boldsymbol{i}}$ | 0.328 | | 0.211 | | 0.658 | | 0.058 | | | -0.531 | | 0.222 | |

# Table S10. The effect of health insurance: results of Heckman two-step model

**(a)**

| **Variable** | **Total hospital expenditure** | | | | | | | | | | | |
| --- | --- | --- | --- | --- | --- | --- | --- | --- | --- | --- | --- | --- |
|  | **Local fund** | | | | **Location of insurance settlement** | | | | **Both** | | | |
|  | **Selection model (hospital admission, n=13065)** | | **Outcome model**  **(n=2186)** | | **Selection model (hospital admission, n=13065)** | | **Outcome model**  **(n=2186)** | | **Selection model (hospital admission, n=13065)** | | **Outcome model**  **(n=2186)** | |
|  | **Coef** | **P** | **Coef** | **P** | **Coef** | **P** | **Coef** | **P** | **Coef** | **P** | **Coef** | **P** |
| **Type of health insurance** (Ref: BMIURR) | | | | | | | | | | | | |
| BMIUE | -0.025 | 0.479 | **0.197** | **<0.001** | 0.294 | <0.001 | 0.078 | 0.191 | 0.020 | 0.625 | **0.127** | **0.011** |
| Free medical care | -0.187 | 0.282 | 0.396 | 0.092 | -0.032 | 0.872 | 0.477 | 0.091 | -0.133 | 0.511 | 0.504 | 0.075 |
| **Local fund** (**Ref**: no) | | | | | | | | | | | | |
| yes | 0.133 | <0.001 | **-0.099** | **0.015** |  |  |  |  | 0.439 | <0.001 | -0.096 | 0.124 |
| **Location of insurance settlement** (Ref: None-residential location) | | | | | | | | | | | | |
| Residential location | |  |  |  |  |  | **-0.196** | **<0.001** |  |  | **-0.126** | **0.012** |
| **Gender** (Ref: Female) | | | | | | | | | | | | |
| Male | -0.277 | <0.001 | **0.171** | **0.005** | -0.270 | <0.001 | **0.173** | **0.002** | -0.268 | <0.001 | **0.145** | **0.007** |
| **Age** (Ref: 15-29 years) | | | | | | | | | | | | |
| 30-44 | -0.441 | <0.001 | 0.057 | 0.490 | -0.387 | <0.001 | 0.053 | 0.428 | -0.386 | <0.001 | 0.018 | 0.784 |
| 45-59 | -0.491 | <0.001 | 0.092 | 0.344 | -0.391 | <0.001 | 0.138 | 0.075 | -0.358 | <0.001 | 0.100 | 0.185 |
| 60 and above | -0.256 | <0.001 | 0.019 | 0.801 | -0.159 | 0.008 | 0.086 | 0.272 | -0.052 | 0.392 | 0.053 | 0.498 |
| **Educational attainment** (Ref: Primary school or below) | | | | | | | | | | | | |
| Junior high | 0.106 | 0.001 | **0.117** | **0.011** | 0.103 | 0.008 | **0.162** | **0.004** | 0.139 | <0.001 | **0.166** | **0.004** |
| ≥Senior high | 0.193 | <0.001 | **0.118** | **0.042** | 0.262 | <0.001 | **0.163** | **0.018** | 0.270 | <0.001 | **0.184** | **0.007** |
| **Marital status** (Ref: Single) | | | | | | | | | | | | |
| Married/ Cohabiting | 1.019 | <0.001 | 0.182 | 0.359 | 0.986 | <0.001 | 0.089 | 0.590 | 1.010 | <0.001 | 0.184 | 0.253 |
| Divorced/ Widowed | 0.816 | <0.001 | -0.070 | 0.699 | 0.812 | <0.001 | -0.173 | 0.321 | 0.823 | <0.001 | -0.084 | 0.628 |
| **Monthly per capita household income ranking** (Ref: Lowest) | | | | | | | | | | | | |
| Lower | -0.037 | 0.254 | 0.044 | 0.286 | -0.059 | 0.141 | 0.070 | 0.204 | -0.045 | 0.262 | 0.062 | 0.252 |
| Middle | -0.056 | 0.104 | 0.064 | 0.152 | -0.035 | 0.409 | 0.069 | 0.224 | -0.01 | 0.814 | 0.066 | 0.239 |
| Higher | -0.036 | 0.318 | 0.082 | 0.071 | -0.042 | 0.344 | **0.127** | **0.030** | -0.019 | 0.678 | **0.120** | **0.040** |
| Highest | -0.027 | 0.502 | **0.177** | **<0.001** | -0.033 | 0.499 | **0.159** | **0.013** | -0.015 | 0.76 | **0.156** | **0.013** |
| **Self-rated health** (Ref: Poor) | | | | | | | | | | | | |
| General | -0.414 | <0.001 | **-0.402** | **<0.001** | -0.378 | <0.001 | **-0.242** | **0.002** | -0.341 | <0.001 | **-0.283** | **<0.001** |
| Good | -0.322 | <0.001 | **-0.591** | **<0.001** | -0.287 | <0.001 | **-0.454** | **<0.001** | -0.261 | <0.001 | **-0.484** | **<0.001** |
| **Distance of migration** (Ref: Inter-county) | | | | | | | | | | | | |
| Inter-city | -0.082 | 0.009 |  |  | -0.135 | <0.001 |  |  | -0.175 | <0.001 |  |  |
| Inter-province | -0.217 | <0.001 |  |  | -0.371 | <0.001 |  |  | -0.428 | <0.001 |  |  |
| **Years of residing in migration destination** (Ref: <1) | | | | | | | | | | | | |
| 1-4 | -0.030 | 0.416 |  |  | 0.126 | 0.009 |  |  | 0.102 | 0.035 |  |  |
| 5-9 | -0.061 | 0.122 |  |  | 0.137 | 0.007 |  |  | 0.102 | 0.047 |  |  |
| 10 and above | -0.049 | 0.230 |  |  | 0.169 | 0.001 |  |  | 0.102 | 0.055 |  |  |
| **Migration destination** (Ref: Pearl River Delta) | | | | | | | | | | | | |
| Yangtze River delta | 0.094 | 0.071 | **0.170** | **0.017** | -0.040 | 0.543 | **0.235** | **0.012** | 0.002 | 0.980 | **0.227** | **0.016** |
| Circum-Bohai Sea | 0.114 | 0.036 | **0.184** | **0.016** | 0.032 | 0.633 | **0.198** | **0.035** | 0.065 | 0.332 | **0.195** | **0.039** |
| others | 0.226 | <0.001 | -0.027 | 0.742 | 0.193 | 0.001 | -0.057 | 0.532 | 0.201 | 0.001 | -0.041 | 0.652 |
| **mills** | **Coef** | | **P** | | **Coef** | | **P** | | **Coef** | | **P** | |
| $\boldsymbol{\lambda}_{\boldsymbol{i}}$ | 0.045 | | 0.852 | | -0.026 | | 0.873 | | 0.095 | | 0.544 | |

**(b)**

| **Variable** | **Out-of-pocket payments** | | | | | | | | | | | |
| --- | --- | --- | --- | --- | --- | --- | --- | --- | --- | --- | --- | --- |
|  | **Local fund** | | | | **Location of insurance settlement** | | | | **Both** | | | |
|  | **Selection model (hospital admission, n=13065)** | | **Outcome model**  **(n=2186)** | | **Selection model (hospital admission, n=13065)** | | **Outcome model**  **(n=2186)** | | **Selection model (hospital admission, n=13065)** | | **Outcome model**  **(n=2186)** | |
|  | **Coef** | **P** | **Coef** | **P** | **Coef** | **P** | **Coef** | **P** | **Coef** | **P** | **Coef** | **P** |
| **Type of health insurance** (Ref: BMIURR) | | | | | | | | | | | | |
| BMIUE | -0.025 | 0.479 | **-0.144** | **0.005** | 0.294 | <0.001 | **-0.267** | **<0.001** | 0.020 | 0.625 | **-0.171** | **0.005** |
| Free medical care | -0.187 | 0.282 | -0.232 | 0.413 | -0.032 | 0.872 | 0.015 | 0.966 | -0.133 | 0.511 | 0.064 | 0.853 |
| **Local fund** (**Ref**: no) | |  |  |  |  |  |  |  |  |  |  |  |
| yes | 0.133 | <0.001 | **-0.237** | **<0.001** |  |  |  |  | 0.439 | <0.001 | **-0.183** | **0.017** |
| **Location of insurance settlement** (Ref: None-residential location) | | | | | | | | | | | | |
| Residential location | |  |  |  |  |  | **-0.391** | **<0.001** |  |  | **-0.262** | **<0.001** |
| **Gender** (Ref: Female) | | | | | | | | | | | | |
| Male | -0.277 | <0.001 | -0.035 | 0.637 | -0.270 | <0.001 | 0.118 | 0.080 | -0.268 | <0.001 | 0.065 | 0.324 |
| **Age** (Ref: 15-29 years) | | | | | | | | | | | | |
| 30-44 | -0.441 | <0.001 | -0.187 | 0.062 | -0.387 | <0.001 | -0.009 | 0.914 | -0.386 | <0.001 | -0.075 | 0.346 |
| 45-59 | -0.491 | <0.001 | **-0.249** | **0.034** | -0.391 | <0.001 | 0.029 | 0.763 | -0.358 | <0.001 | -0.045 | 0.624 |
| 60 and above | -0.256 | <0.001 | **-0.313** | **<0.001** | -0.159 | 0.008 | -0.057 | 0.556 | -0.052 | 0.392 | -0.120 | 0.211 |
| **Educational attainment** (Ref: Primary school or below) | | | | | | | | | | | | |
| Junior high | 0.106 | 0.001 | **0.132** | **0.017** | 0.103 | 0.008 | **0.188** | **0.006** | 0.139 | <0.001 | **0.195** | **0.005** |
| ≥Senior high | 0.193 | <0.001 | **0.149** | **0.034** | 0.262 | <0.001 | **0.180** | **0.032** | 0.270 | <0.001 | **0.221** | **0.008** |
| **Marital status** (Ref: Single) | | | | | | | | | | | | |
| Married/ Cohabiting | 1.019 | <0.001 | **0.637** | **0.008** | 0.986 | <0.001 | 0.176 | 0.382 | 1.010 | <0.001 | 0.359 | 0.068 |
| Divorced/ Widowed | 0.816 | <0.001 | 0.237 | 0.278 | 0.812 | <0.001 | -0.160 | 0.456 | 0.823 | <0.001 | 0.010 | 0.961 |
| **Monthly per capita household income ranking** (Ref: Lowest) | | | | | | | | | | | | |
| Lower | -0.037 | 0.254 | 0.028 | 0.582 | -0.059 | 0.141 | 0.055 | 0.412 | -0.045 | 0.262 | 0.041 | 0.539 |
| Middle | -0.056 | 0.104 | 0.022 | 0.683 | -0.035 | 0.409 | 0.037 | 0.596 | -0.010 | 0.814 | 0.031 | 0.653 |
| Higher | -0.036 | 0.318 | 0.074 | 0.181 | -0.042 | 0.344 | **0.142** | **0.048** | -0.019 | 0.678 | 0.127 | 0.075 |
| Highest | -0.027 | 0.502 | **0.182** | **0.003** | -0.033 | 0.499 | 0.136 | 0.081 | -0.015 | 0.760 | 0.131 | 0.091 |
| **Self-rated health** (Ref: Poor) | | | | | | | | | | | | |
| General | -0.414 | <0.001 | **-0.525** | **<0.001** | -0.378 | <0.001 | **-0.221** | **0.019** | -0.341 | <0.001 | **-0.300** | **0.001** |
| Good | -0.322 | <0.001 | **-0.645** | **<0.001** | -0.287 | <0.001 | **-0.446** | **<0.001** | -0.261 | <0.001 | **-0.505** | **<0.001** |
| **Distance of migration** (Ref: Inter-county) | | | | | | | | | | | | |
| Inter-city | -0.082 | 0.009 |  |  | -0.135 | <0.001 |  |  | -0.175 | <0.001 |  |  |
| Inter-province | -0.217 | <0.001 |  |  | -0.371 | <0.001 |  |  | -0.428 | <0.001 |  |  |
| **Years of residing in migration destination** (Ref: <1) | | | | | | | | | | | | |
| 1-4 | -0.030 | 0.416 |  |  | 0.126 | 0.009 |  |  | 0.102 | 0.035 |  |  |
| 5-9 | -0.061 | 0.122 |  |  | 0.137 | 0.007 |  |  | 0.102 | 0.047 |  |  |
| 10 and above | -0.049 | 0.230 |  |  | 0.169 | 0.001 |  |  | 0.102 | 0.055 |  |  |
| **Migration destination** (Ref: Pearl River Delta) | | | | | | | | | | | | |
| Yangtze River delta | 0.094 | 0.071 | **0.361** | **<0.001** | -0.040 | 0.543 | **0.537** | **<0.001** | 0.002 | 0.980 | **0.520** | **<0.001** |
| Circum-Bohai Sea | 0.114 | 0.036 | **0.368** | **<0.001** | 0.032 | 0.633 | **0.471** | **<0.001** | 0.065 | 0.332 | **0.465** | **<0.001** |
| others | 0.226 | <0.001 | **0.213** | **0.029** | 0.193 | 0.001 | 0.207 | 0.065 | 0.201 | 0.001 | **0.238** | **0.032** |
| **mills** | **Coef** | | **P** | | **Coef** | | **P** | | **Coef** | | **P** | |
| $\boldsymbol{\lambda}_{\boldsymbol{i}}$ | 0.591 | | 0.041 | | -0.072 | | 0.724 | | 0.161 | | 0.400 | |

**(c)**

| **Variable** | **Reimbursement ratio of hospital expenditure** | | | | | | | | | | | |
| --- | --- | --- | --- | --- | --- | --- | --- | --- | --- | --- | --- | --- |
|  | **Local fund** | | | | **Location of insurance settlement** | | | | **Both** | | | |
|  | **Selection model (insurance reimbursement, n=3186)** | | **Outcome model**  **(n=2229)** | | **Selection model (insurance reimbursement, n=3186)** | | **Outcome model**  **(n=2229)** | | **Selection model (insurance reimbursement, n=3186)** | | **Outcome model**  **(n=2229)** | |
|  | **Coef** | **P** | **Coef** | **P** | **Coef** | **P** | **Coef** | **P** | **Coef** | **P** | **Coef** | **P** |
| **Type of health insurance** (Ref: BMIURR) | | | | | | | | | | | | |
| BMIUE | 0.320 | <0.001 | **0.141** | **<0.001** | 0.688 | <0.001 | **0.141** | **<0.001** | 0.320 | <0.001 | **0.147** | **<0.001** |
| Free medical care | 5.954 | >0.99 | **0.250** | **0.002** | 5.876 | >0.99 | **0.268** | **0.002** | 5.954 | >0.99 | **0.290** | **<0.001** |
| **Local fund** (Ref: no) | |  |  |  |  |  |  |  |  |  |  |  |
| yes | 0.574 | <0.001 | **0.069** | **0.024** |  |  |  |  | 0.574 | <0.001 | 0.047 | 0.121 |
| **Location of insurance settlement** (Ref: None-residential location) | | | | | |  |  |  |  |  |  |  |
| Residential location | |  |  |  |  |  | **0.092** | **<0.001** |  |  | **0.066** | **<0.001** |
| **Gender** (Ref: Female) | | | | | | | | | | | | |
| Male | 0.025 | 0.670 | **0.031** | **0.004** | 0.005 | 0.930 | **0.029** | **0.006** | 0.025 | 0.670 | **0.031** | **0.004** |
| **Age** (Ref: 15-29 years) | | | | | | | | | | | | |
| 30-44 | 0.090 | 0.164 | **0.033** | **0.019** | 0.095 | 0.138 | 0.028 | 0.057 | 0.090 | 0.164 | **0.033** | **0.017** |
| 45-59 | 0.356 | <0.001 | 0.043 | 0.087 | 0.313 | <0.001 | 0.038 | 0.129 | 0.356 | <0.001 | **0.052** | **0.039** |
| 60 and above | 0.549 | <0.001 | 0.057 | 0.096 | 0.445 | <0.001 | 0.042 | 0.171 | 0.549 | <0.001 | **0.066** | **0.05** |
| **Educational attainment** (Ref: Primary school or below) | | | | | | | | | | | | |
| Junior high | 0.094 | 0.202 | -0.024 | 0.097 | 0.045 | 0.539 | -0.025 | 0.076 | 0.094 | 0.202 | -0.021 | 0.136 |
| ≥Senior high | 0.210 | 0.009 | -0.026 | 0.161 | 0.189 | 0.019 | -0.026 | 0.132 | 0.210 | 0.009 | -0.019 | 0.282 |
| **Marital status** (Ref: Single) | | | | | | | | | | | | |
| Married/ Cohabiting | 0.116 | 0.386 | **-0.069** | **0.012** | 0.107 | 0.419 | **-0.066** | **0.016** | 0.116 | 0.386 | **-0.062** | **0.021** |
| Divorced/ Widowed | 0.250 | 0.220 | -0.039 | 0.302 | 0.277 | 0.167 | -0.029 | 0.451 | 0.250 | 0.220 | -0.025 | 0.503 |
| **Monthly per capita household income ranking** (Ref: Lowest) | | | | | | | | | | | | |
| Lower | 0.002 | 0.982 | 0.003 | 0.847 | -0.010 | 0.882 | 0.001 | 0.920 | 0.002 | 0.982 | 0.002 | 0.912 |
| Middle | 0.137 | 0.073 | 0.002 | 0.906 | 0.121 | 0.109 | 0.005 | 0.764 | 0.137 | 0.073 | 0.008 | 0.600 |
| Higher | 0.011 | 0.890 | -0.001 | 0.970 | -0.006 | 0.935 | -0.002 | 0.871 | 0.011 | 0.890 | -0.002 | 0.918 |
| Highest | 0.025 | 0.773 | -0.004 | 0.827 | 0.004 | 0.958 | -0.001 | 0.928 | 0.025 | 0.773 | -0.002 | 0.919 |
| **Self-rated health** (Ref: Poor) | | | | | | | | | | | | |
| General | -0.111 | 0.204 | 0.008 | 0.604 | -0.144 | 0.096 | 0.006 | 0.708 | -0.111 | 0.204 | 0.005 | 0.759 |
| Good | -0.281 | 0.001 | 0.015 | 0.481 | -0.300 | 0.001 | 0.013 | 0.564 | -0.281 | 0.001 | 0.006 | 0.789 |
| **Distance of migration** (Ref: Inter-county) | | | | | | | | | | | | |
| Inter-city | -0.219 | 0.001 | -0.016 | 0.302 | -0.164 | 0.013 | 0.005 | 0.703 | -0.219 | 0.001 | -0.009 | 0.562 |
| Inter-province | -0.484 | <0.001 | -0.018 | 0.504 | -0.406 | <0.001 | 0.012 | 0.630 | -0.484 | <0.001 | -0.016 | 0.552 |
| **Years of residing in migration destination** (Ref: <1) | | | | | | | | | | | | |
| 1-4 | -0.010 | 0.909 |  |  | 0.017 | 0.837 |  |  | -0.010 | 0.909 |  |  |
| 5-9 | 0.078 | 0.394 |  |  | 0.111 | 0.217 |  |  | 0.078 | 0.394 |  |  |
| 10 and above | 0.015 | 0.880 |  |  | 0.117 | 0.220 |  |  | 0.015 | 0.880 |  |  |
| **Migration destination** (Ref: Pearl River Delta) | | | | | | | | | | | | |
| Yangtze River delta | -0.256 | 0.037 | **-0.066** | **0.017** | -0.322 | 0.008 | **-0.065** | **0.027** | -0.256 | 0.037 | **-0.073** | **0.007** |
| Circum-Bohai Sea | -0.421 | 0.001 | **-0.068** | **0.036** | -0.448 | <0.001 | **-0.071** | **0.035** | -0.421 | 0.001 | **-0.082** | **0.010** |
| others | -0.046 | 0.676 | **-0.072** | **0.001** | -0.053 | 0.630 | **-0.077** | **<0.001** | -0.046 | 0.676 | **-0.077** | **<0.001** |
| **mills** | **Coef** | | **P** | | **Coef** | | **P** | | **Coef** | | **P** | |
| $\boldsymbol{\lambda}_{\boldsymbol{i}}$ | -0.078 | | 0.473 | | -0.078 | | 0.494 | | -0.009 | | 0.937 | |
